# Chemistry of CS_2_ and CS_3_ Bridged Decaborane Analogues: Regular Coordination Versus Cluster Expansion

**DOI:** 10.3390/molecules28030998

**Published:** 2023-01-19

**Authors:** Ketaki Kar, Suvam Saha, Rahul Maganbhai Parmar, Arindam Roy, Marie Cordier, Thierry Roisnel, Sundargopal Ghosh

**Affiliations:** 1 Department of Chemistry, Indian Institute of Technology Madras, Chennai 600036, India; 2 Univ Rennes, CNRS, Institut des Sciences Chimiques de Rennes, UMR 6226, F-35000 Rennes, France

**Keywords:** metallaheteroborane, fusion, coordination, expansion, cluster

## Abstract

In an effort to synthesize metallaheteroborane clusters of higher nuclearity, the reactivity of metallaheteroboranes, *nido*-[(Cp*M)_2_B_6_S_2_H_4_(CS_3_)] (Cp* = C_5_Me_5_) (**1**: M = Co; **2**: M = Rh) with various metal carbonyls have been investigated. Photolysis of *nido*-**1** and *nido*-**2** with group 6 metal carbonyls, M’(CO)_5_.THF (M’ = Mo or W) were performed that led to the formation of a series of adducts [(Cp*M)_2_B_6_S_2_H_4_(CS_3_){M’(CO)_5_}] (**3**: M = Co, M’ = Mo; **4**: M = Co, M’ = W; **5**: M = Rh, M’ = Mo; **6**: M = Rh, M’ = W) instead of cluster expansion reactions. In these adducts, the S atom of C=S group of di(thioboralane)thione {B_2_CS_3_} moiety is coordinated to M’(CO)_5_ (M = Mo or W) in *η*^1^-fashion. On the other hand, thermolysis of *nido*-**1** with Ru_3_(CO)_12_ yielded one fused metallaheteroborane cluster [{Ru(CO)_3_}_3_S{Ru(CO)}{Ru(CO)_2_}Co_2_B_6_SH_4_(CH_2_S_2_){Ru(CO)_3_}_2_S], **7**. This 20-vertex-fused cluster is composed of two tetrahedral {Ru_3_S} and {Ru_2_B_2_}, a flat butterfly {Ru_3_S} and one octadecahedron {Co_2_RuB_7_S} core with one missing vertex, coordinated to {Ru_2_SCH_2_S_2_} through two boron and one ruthenium atom. On the other hand, the room temperature reaction of *nido*-**2** with Co_2_(CO)_8_ produced one 19-vertex fused metallaheteroborane cluster [(Cp*Rh)_2_B_6_H_4_S_4_{Co(CO)}_2_{Co(CO)_2_}_2_(*μ*-CO)S{Co(CO)_3_}_2_], **8**. Cluster **8** contains one *nido*-decaborane {Rh_2_B_6_S_2_}, one butterfly {Co_2_S_2_} and one bicapped square pyramidal {Co_6_S} unit that exhibits an intercluster fusion with two sulfur atoms in common. Clusters **3**–**6** have been characterized by multinuclear NMR and IR spectroscopy, mass spectrometry and structurally determined by XRD analyses. Furthermore, the DFT calculations have been carried out to gain insight into electronic, structural and bonding patterns of the synthesized clusters.

## 1. Introduction

**Chart 1:**
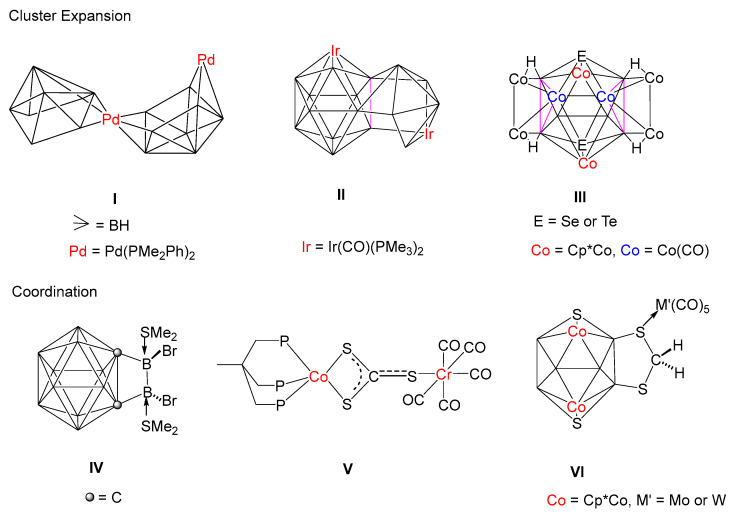
Different types of cluster expansion and coordination.

The chemistry of polyhedral boron clusters has been significantly developed owing to their noteworthy structural appeal exhibited by different coordination modes, unique bonding patterns and their extensive applications in catalysis, material science, boron neutron capture therapy (BNCT), etc [1,2,3,4,5,6,7]. Starting with Hawthorne and followed by many main group pioneers such as Lipscomb [8], Grimes [9], Welch [10], Kennedy [11], Fehlner [12,13] and Xie [14], we [15,16,17,18,19,20,21,22,23,24,25,26,27,28] and others [29,30,31] have reported numerous single cages as well as condensed polyhedral clusters containing boron, chalcogens, carbon and transition metal fragments. Due to the deficiency of the electron octet around boron atom, these clusters display some unusual bonding features with main group elements as well as transition metals [32,33]. Molecular assemblies that involve two or more individual single clusters closely joined together are called fused clusters. Such fused clusters species are known as “macropolyhedral” species that are formed by the fusion of smaller building blocks like tetrahedrons, trigonal bipyramids, octahedrals, etc [34,35,36]. This fusion can occur by sharing common atoms, edges and faces (Chart 1). For example, Kennedy et al., have synthesized **I** from thermal autofusion of [(Pme_2_Ph)_2_PdB_8_H_12_] [37]. **I** is a vertex-fused cluster which can be contemplated as simple convergence of two starting materials of *arachno* {MB_8_} geometries. They have also isolated an 18-vertex macropolyhedral diiridaborane(**II**), comprised of one 12-vertex *closo*-{IrB_11_} and one 8-vertex *nido*-{IrB_7_}, fused by a common {B_2_} edge [38]. Successively, we and Fehlner have developed synthetic strategies for the isolation of fused clusters utilizing preformed metallaboranes and monobornes or metal carbonyls [39,40,41,42,43]. For example, the reaction of [(Cp*ReH_2_)_2_B_4_H_4_] with [Co_2_(CO)_8_] led to the formation of a 6 SEP hypoelectronic cluster [(Cp*Re)_2_Co_2_(CO)_5_B_4_H_4_], in which a 4-electron {Co_2_(CO)_5_} fragment is coplanar with the B_4_ fragment [39]. Fehlner has isolated a hybrid cluster [Fe_2_(CO)_6_(*η*^5^-C_5_Me_5_RuCO)(*η*^5^-C_5_Me_5_Ru)B_6_H_10_] from the reaction of *nido*-[(*η*^5^-C_5_Me_5_Ru)_2_B_6_H_12_] and [Fe_2_(CO)_9_], in which a {Fe_2_B_2_} tetrahedron has been fused to ruthenaborane moiety through two common boron atoms [40]. Recently, we have reported an unusual doubly face-fused 16-vertex macropolyhedral cluster, [(Cp*Co)_2_ B_6_H_6_E_2_{Co_2_(CO)_2_}(*μ*-CO)_2_{Co_4_(CO)_8_}] (E = Se or Te), **III** which can be described as a fusion of three individual olyhedral such as the two 5-vertex *nido*-square pyramid {Co_3_B_2_} and one 12-vertex icosahedron {Co_4_B_6_E_2_} units having two common triangular faces [43].

Apart from cluster fusion, several coordination compounds have been synthesized involving metal carbonyl fragments, chalcogens, boranes, and metallaboranes following the same way [44,45,46]. Beginning from the synthesis of [Pt(CS_2_)(PPh_3_)_2_], the first CS_2_ metal complex by Wilkinson and co-workers, a range of CS_2_ complexes containing transition metals have been synthesized [47]. Transition metal-{S_2_CS} moiety can act as a ligand to bind to one or two metal centres [48]. As shown in Chart 1, Zouwei Xie and co-workers have reported [1,2-{BbrSMe_2_}_2_-*o*-C_2_B_10_H_10_] (**IV**) in which Sme_2_ groups are coordinated to *exo*-boron atoms [49]. Bianchini et al., have reported complex **V** in which two sulfur atoms are connected to Co, whereas the third sulfur atom of {CS_3_} unit is linked to a Cr(CO)_5_ fragment through a *σ* bond [50]. Recently, we have isolated [(Cp*Co)_2_B_6_S_2_H_4_(CH_2_S_2_) {M’(CO)_5_}] (**VI**, M’ = Mo, W) from the photolysis of {CH_2_S_2_} bridged decaborane-14 analogue *nido*-[(Cp*Co)_2_B_6_S_2_H_4_(CH_2_S_2_)] with {M’(CO)_5_·THF} (M’ = Mo or W) fragments [42]. The molecular structure of **VI** shows that one of the sulfur atoms of the {CH_2_S_2_} moiety is coordinated to {M’(CO)_5_} (M’ = Mo or W) in *η*^1^-fashion. However, when *nido*-[(Cp*Co)_2_ B_6_S_2_H_4_(CH_2_S_2_)] was treated with [Fe_2_(CO)_9_] at room temperature, it afforded an 11-vertex *nido*-[(Cp*Co)_2_B_6_S_2_H_4_(CH_2_S_2_){Fe(CO)_3_}], which is similar to [C_2_B_9_H_11_]^2−^ with a five-membered pentahapto coordinating face [43]. Along with *nido*-[(Cp*Co)_2_B_6_S_2_H_4_(CH_2_S_2_)], we also have isolated CS_3_ bridged decaborane-14 analogues, *nido*-[(Cp*M)_2_B_6_S_2_H_4_(CS_3_)] (**1**: M = Co; **2**: M = Rh) [42]. Based on the computational studies, it was proposed that clusters *nido-***1** and *nido-***2** have the ability to attract electrophiles as well as nucleophiles. Thus, in order to validate our theoretical assumptions, we have investigated the reactivity of *nido*-**1** and *nido*-**2** with different metal carbonyls such as M’(CO)_5_.THF (M’ = Mo or W), Ru_3_(CO)_12_ and Co_2_(CO)_8_ that afforded coordination compounds as well as higher-vertex fused macropolyhedral clusters with unusual bonding.

## 2. Results and Discussion

### 2.1. Reactivity of nido-***1*** and nido-***2*** with M’(CO)_5_.THF (M = Mo, W)

**Scheme 1:**
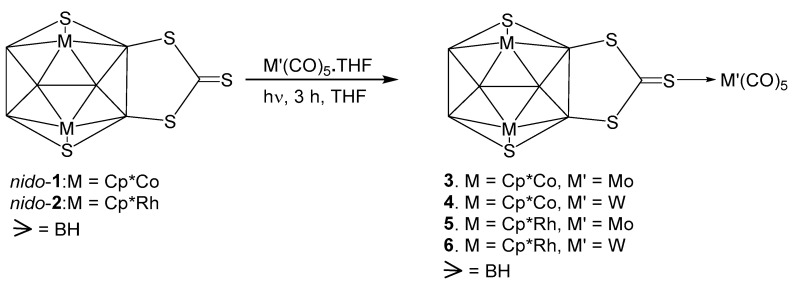
Synthesis of metal carbonyl coordinated clusters **3**, **4**, **5** and **6**.

As shown in Scheme 1, photolysis of *nido*-**1** with M’(CO)_5_.THF (M’ = Mo or W) produced adducts [(Cp*Co)_2_B_6_S_2_H_4_(CS_3_){M’(CO)_5_}] (**3**: M’ = Mo; **4**: M’ = W) as violet and red solids, respectively (Scheme 1). These compounds were characterized by ^1^H{^11^B}, ^11^B{^1^H}, and IR spectroscopy along with ESI mass spectrometry. The ^1^H{^11^B} NMR spectra of **3** and **4** exhibit the existence of single Cp* environments appearing at *δ* = 1.65 and 1.69 ppm, respectively. The ^11^B{^1^H} NMR spectra of **3** and **4** display four resonances appeared at *δ* = 26.5, 27.6, 33.0 and 37.7 ppm (for **3**) and *δ* = 24.6, 26.4. 33.4 and 38.2 ppm (for **4**). However, the identity was unclear until an X-ray crystallographic analysis was carried out for one of them. Despite of our several attempts, we could not get good quality crystals for **3**. The CH_2_Cl_2_-hexane solution of **4** at −5 °C yielded X-ray quality crystals that allowed us to study the X-ray diffraction of **4**.

**Figure 1:**
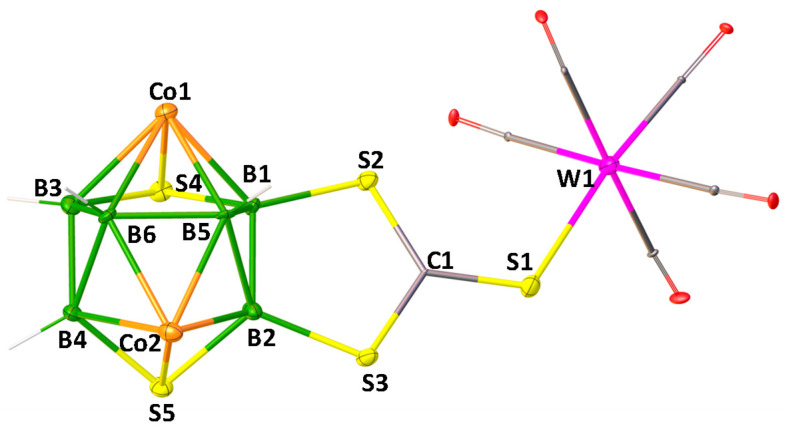
Molecular structure and labelling diagram of **4** (note that Cp* ligands attached to metals are omitted for clarity). Selected bond lengths (Å) and angles (°). **4**: B1−B2 1.790(19), W1−S1 2.529(3), S1−C1 1.648(12), S2−B1 1.886(12); C1−S1−W1 116.3(4), C1−S2−B1 102.0(6).

The solid-state X-ray structure of **4**, shown in Figure 1, reveals that the S atom of C=S group of di(thioboralane)-thione {B_2_CS_3_} moiety is coordinated to W(CO)_5_ in *η*^1^-fashion. The S1-C1 bond distance of 1.648(12) Å in **4** is slightly longer than C=S bond length of *nido*-**1** [1.636(6) Å]. The W-S bond distance of **4** [W1-S1 2.529 (3) Å] is slightly longer compared to [CpW(CO)_2_{*η*^2^-(S_2_CC_6_H_4_Me_4_)}] (2.472 Å) [51]. This may be due to the effect of monodentate ligation of {CS_3_} to W atom. Deliberating the spectroscopic data of **3** and **4** along with the X-ray structure of **4**, it is reasonable to assume that **3** is a Mo analogue of **4**.

**Figure 2:**
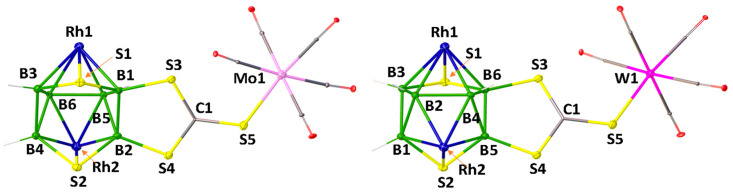
Molecular structures and labelling diagrams of **5** and **6** (note that Cp* ligands attached to metals are omitted for clarity). Selected bond lengths (Å) and angles (°): **5**: B1−B2 1.808(8), Mo1−S5 2.5388(15), S5−C1 1.668(6), S3−B1 1.869(6); C1−S5−Mo1 117.1(2), C1−S3−B1 101.1(3). **6**: B5−B6 1.809(4), W1−S5 2.5293(7), S5−C1 1.672(3), S3−B6 1.873(3); C1−S5−W1 116.73(9), C1−S3−B6 101.03(12).

Similarly, compounds **5** and **6** were synthesized from the reaction of *nido*-**2** with M’(CO)_5_.THF (M’ = Mo or W) at photolytic conditions, respectively. The ^1^H{^11^B} NMR spectra of **5** and **6** exhibit the existence of single Cp* environments appearing at *δ* = 1.85 and 1.80 ppm, respectively. The ^11^B{^1^H} NMR spectra of **5** and **6** show four resonances each appeared at *δ* = 17.3, 20.3, 24.6 and 30.4 ppm (for **5**) and *δ* = 16.7, 20.5, 23.9 and 24.9 ppm (for **6**). All these spectroscopic data of **5** and **6** suggest that they are analogous to **3** and **4**, respectively. In order to confirm this assumption, the single-crystal X-ray structure analyses of suitable crystals of **5** and **6** were undertaken. As shown in Figure 2, the solid-state structures of **5** and **6** display that S atom of C=S group of di(thioboralane)-thione {B_2_CS_3_} moiety is coordinated to M’(CO)_5_ (M’ = Mo or W) in *η*^1^-fashion. The C=S bond distance of 1.668(6) Å in **5** and 1.672 (3) Å in **6** are slightly longer than the C=S bond length of *nido*-**2** [1.635(9) Å]. The M’-S bond distances of **5** [Mo1-S5 2.539 (15) Å] and **6** [W1–S5 2.293 (7) Å] are slightly longer compared to those of [CpMo(CO)_2_{*η*^2^-(S_2_CCH_2_*^t^*Bu)}] (2.477 Å) and [CpW(CO)_2_{*η*^2^-(S_2_CC_6_H_4_Me_4_)}] (2.472 Å), respectively.[52] This may be due to the effect of monodentate ligation of {CS_3_} to Mo or W atoms.

**Figure 3:**
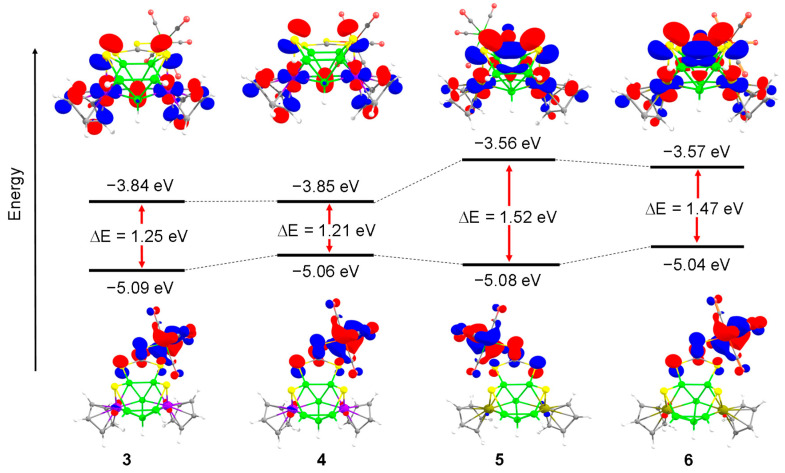
Comparison of frontier molecular orbitals of **3**, **4**, **5** and **6**; respectively. Contour values for isosurface are ±0.04 (e/bohr^3^)^1/2^.

To provide some insight into the electronic structures and bonding relationship of the adducts **3**–**6**, the DFT calculations using the Gaussian 16 program with a BP86/def2-svp level of theory have been carried out. The MO analysis, shown in Figure 3, shows that the HOMO-LUMO gap for **3**, in which the [Mo(CO)_5_] moiety is coordinated to C=S group of di(thioboralane)-thione {B_2_CS_3_} is more as compared to its tungsten analogue **4**. The similar trend has been observed in case of **5** and **6**. However, the HOMO-LUMO gap for the Rh systems (**5** and **6**) is greater than the Co systems (**3** and **4**). The highest HOMO-LUMO gap is observed for compound **5**, which indirectly indicates higher stability than its Co analogue.

### 2.2. Condensed Clusters: [{Ru(CO)_3_}_3_S{Ru(CO)}{Ru(CO)_2_}Co_2_B_6_SH_4_{Ru(CO)_3_}_2_(SCH_2_S_2_)], ***7*** and [(Cp*Rh)_2_B_6_H_4_S_4_{Co(CO)}_2_{Co(CO)_2_}_2_(μ-CO)S{Co(CO)_3_}_2_], ***8***

**Scheme 2:**
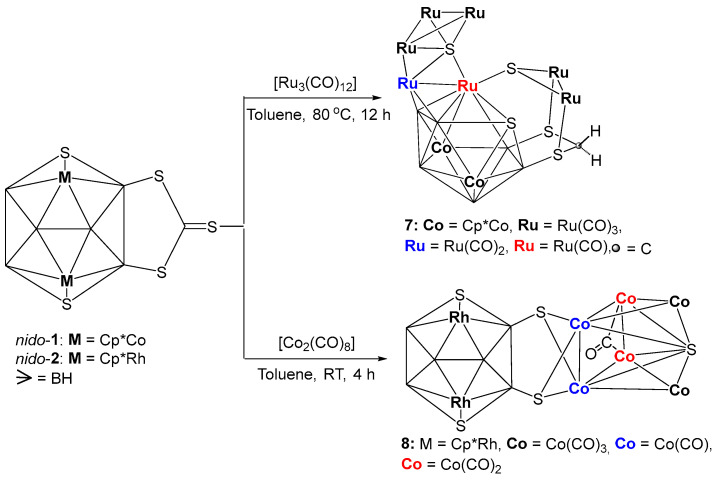
Synthesis of fused clusters **7** and **8**.

Although the reactions of *nido*-**1** and *nido*-**2** with group 6 metal carbonyls yielded simple coordination compounds, reactions with both Ru_3_(CO)_12_ and Co_2_(CO)_8_ led to the formation of condensed clusters [{Ru(CO)_3_}_3_S{Ru(CO)}{Ru(CO)_2_}Co_2_B_6_SH_4_{Ru(CO)_3_}_2_ (SCH_2_S_2_)] **7** and [(Cp*Rh)_2_B_6_H_4_S_4_{Co(CO)}_2_{Co(CO)_2_}_2_(*μ*-CO)S{Co(CO)_3_}_2_] **8,** respectively (Scheme 2). Note that, both these clusters were isolated in very poor yields which were merged with unreacted precursors *nido*-**1** and *nido*-**2** while doing thin-layered chromatography (TLC). Although we were not able to purify them, fractional crystallization allowed us to get a few crystals of them for X-ray diffraction studies. Therefore, the characterization of both **7** and **8** are solely based on the molecular structures obtained from the single crystal XRD analysis.

#### 2.2.1. Structural Account of **7**

**Figure 4:**
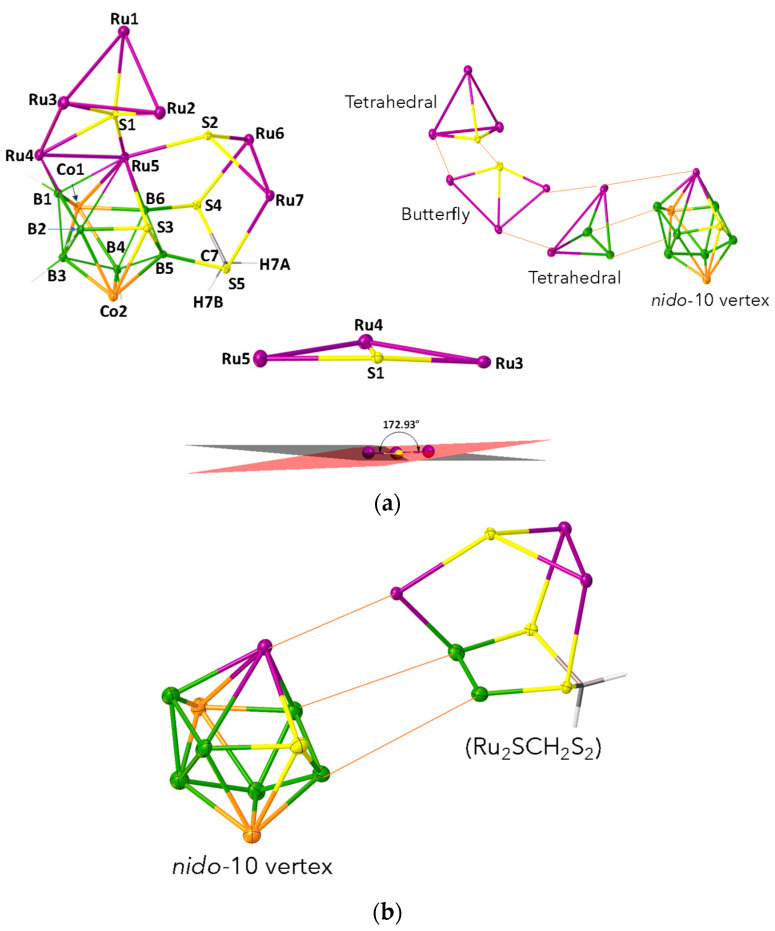
(**a**) Molecular structure, labelling diagram (**left**), fusion demonstration (**right**) and demonstration with dihedral angles between planes of Ru5−S1−Ru4 and Ru4−S1−Ru3 triangles of {Ru_3_S} butterfly subcluster (**bottom**), of **7** (note that Cp* ligands and carbonyl groups attached to metals are omitted for clarity). Selected bond lengths (Å) and angles (°): **7**: Ru1−S1 2.3488(18), Ru3−S1 2.4798(18), Ru4−S1 2.5153(18), Ru3−Ru4 3.0572(8), Ru7−S2 2.4311(17), S5−C7 1.821(7); Ru5−S1−Ru3 145.51(8), Ru4−S1−Ru3 75.47(5). (**b**)Fusion of *nido*-10 vertex {Co_2_B_6_RuS} and {Ru_2_SCH_2_S_2_} cores of **7**. (Cp* ligands and carbonyl groups attached to metals are omitted for clarity).

As shown in Figure 4a, the molecular structure of **7** reveals the molecular formula as [{Ru(CO)_3_}_3_S{Ru(CO)}{Ru(CO)_2_}Co_2_B_6_SH_4_{Ru(CO)_3_}_2_(SCH_2_S_2_)]. This is a 20-vertex macro-polyhedral metallaheteroborane cluster, in which fusion and coordination are observed concurrently (Figure 4a). The cluster **7** comprises of two tetrahedral clusters {Ru_3_S} and {Ru_2_B_2_}, one butterfly {Ru_3_S}, one missing vertex octadecahedron {Co_2_B_6_RuS} and one exopolyhedral moiety {Ru_2_SCH_2_S_2_}. Tetrahedron {Ru_3_S} unit and butterfly {Ru_3_S} unit are fused by one common {Ru-S} edge. The butterfly {Ru_3_S} unit is fused through a common {Ru-Ru} edge with another tetrahedral {Ru_2_B_2_}, fused with one missing vertex octadecahedron {Co_2_B_6_RuS} with a common triangular {RuB_2_} face. The exopolyhedral moiety {Ru_2_SCH_2_S_2_} is coordinated to Ru5, B6 and B5 atoms of *nido*-{Co_2_B_6_RuS} subcluster through its S2, S4 and S5 atoms, respectively (Figure 4b). In other way, we can explain that S2, S4 and S5 atoms of {Ru_2_S(CH_2_S_2_)} unit bridge to Ru5, B6 and B5 atoms of *nido*-{Co_2_B_6_RuS} unit, respectively in *exo* fashion. Additionally, the butterfly {Ru_3_S} unit of **7** is almost flat with a dihedral angle of 172.93° (Figure 4a). Although there are few instances of tetrametallic butterfly clusters that are flattened or almost flattened, flat butterfly clusters containing transition metal and main group vertices are exceptional [52]. The ruthenium-sulfur bond lengths in {Ru_3_S} butterfly core of **7** (avg. 2.489 Å) are considerably longer than the bond length of [(Cp*Ru)_2_(μ,*η*^3^-CHS)_2_] (2.3733 Å) [53,54].

**Figure 5:**
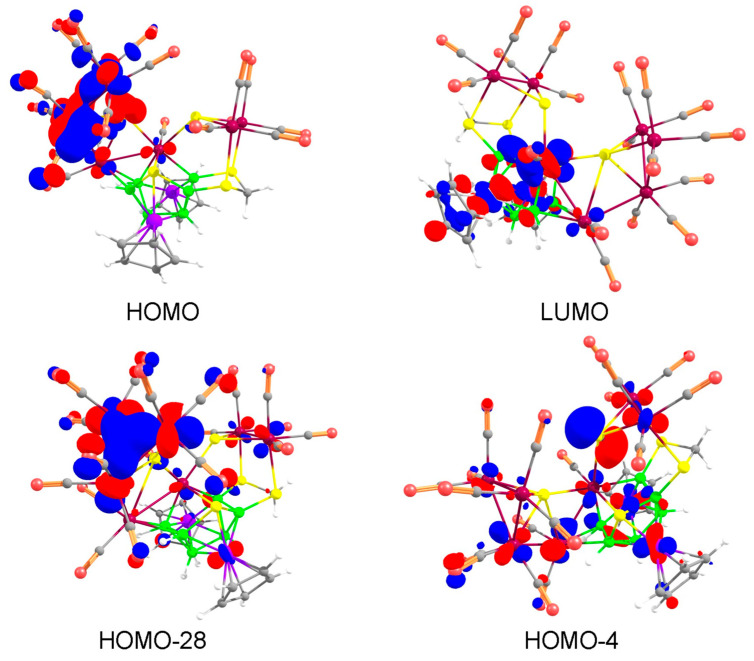
Key frontier molecular orbitals of **7**. Contour values for isosurface are ±0.04 (e/bohr^3^)^1/2^.

The HOMO of **7** exhibits the *π*-bonding interactions among the d orbitals of three Ru atoms and p orbital of one sulfur atom of the {Ru_3_S} tetrahedron core. On the other hand, the σ-antibonding interactions between the Co, Ru (d orbitals) and S atom (p orbital) of the *nido*-{Co_2_B_6_RuS} core was observed in LUMO of **7** (Figure 5). The HOMO-28 shows an extended overlap of the d orbitals of three Ru atoms of the {Ru_3_S} tetrahedron indicating a trimetallic-bonding scenario (Figure 5). The presence of localised non-bonding p orbitals on S2 and S5 atoms observed in HOMO-4 (Figure 5) and HOMO-36 (Figure S23) makes **7** a suitable candidate for further cluster growth reactions.

#### 2.2.2. Structural Account of **8**

**Figure 6:**
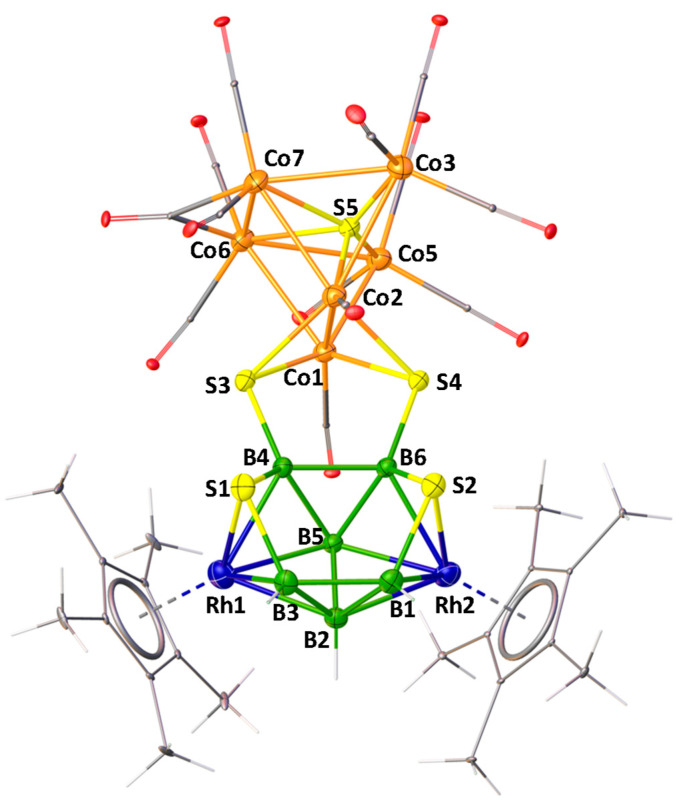
Molecular structure and labelling diagram of **8**. Selected bond lengths (Å) and angles (°). **8**: Co1−Co2 2.4288(6), Co1−S5 2.2790(8), Co1−Co6 2.5962(5), Co6−Co7 2.6164(7), B4−S3 1.868(3), Co1−S3 2.2313(9); Co1−S5-Co2 64.63(2), Co3−S5-Co5 137.88(4).

As shown in Figure 6, the X-ray structure of **8** shows the molecular formula as [(Cp*Rh)_2_B_6_H_4_S_4_{Co(CO)}_2_{Co(CO)_2_}_2_(*μ*-CO)S{Co(CO)_3_}_2_]. Although, this looks like a fusion of three polyhedra, for example, one bicapped square pyramid {Co_6_S}, one butterfly {Co_2_S_2_} and one 10 vertex *nido*-{Rh_2_B_6_S_2_}, careful evaluation of this cluster shows that one bicapped square pyramidal {Co_6_S} core is fused with a butterfly {Co_2_S_2_} unit via one common {Co_2_} edge. This entire fused cluster {S_2_Co_6_S} is coordinated to B4 and B6 atoms of 10 vertex *nido*-{Rh_2_B_6_S_2_} through the wingtip S3 and S4 atoms of the butterfly {Co_2_S_2_}.

**Figure 7:**
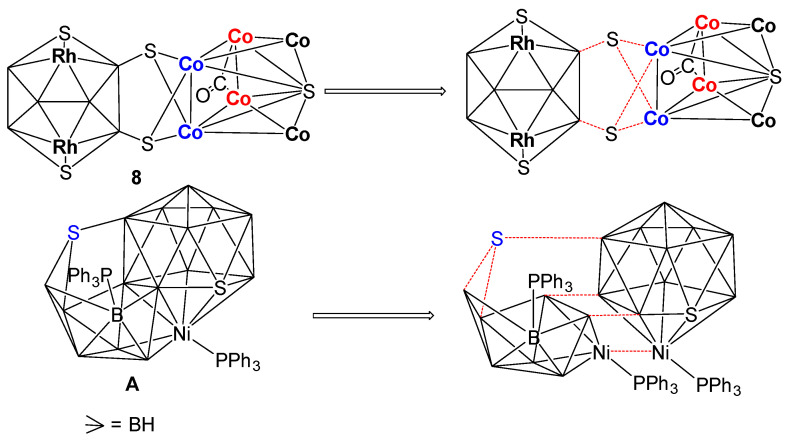
Representation of intercluster crosslink in clusters **8** and [(PPh_3_)NiS_2_B_16_H_12_(PPh_3_)].

The structural account of **8** can also be described in a different approach. As shown in Figure 7, this may be considered as two intercluster crosslinks, in which two sulfur atoms (S3 and S4) are bridged by two Co1 and Co2 atoms of the bicapped square pyramidal {Co_6_S} and two boron atoms (B4 and B6) on the 10-vertex *nido*-{Rh_2_B_6_S_2_} subcluster in *µ*^3^-fashion. A similar description has also been proposed for [(PPh_3_)NiS_2_B_16_H_12_(PPh_3_)] by Kennedy [55]. As shown in Figure 7, in case of **A**, there is an intercluster crosslink in which a sulfur atom bridges two boron atoms of the 9-vertex *nido*-{NiB_8_} subcluster and a boron atom on the 12-vertex *closo*-{NiSB_10_} subcluster in *µ*^3^ fashion.

**Figure 8:**
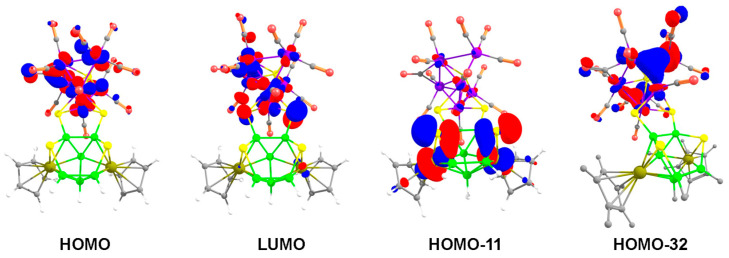
Key frontier molecular orbitals of **8**. Contour values for isosurface are ±0.04 (e/bohr^3^)^1/2^.

As shown in Figure 8, the MO analysis of cluster **8** reveals that the HOMO consists of mainly the d orbitals of cobalt atoms of {Co_6_S} core, whereas the non-bonding p-orbitals of the wingtip S3 and S4 atoms of the butterfly {Co_2_S_2_} contribute to the LUMO. The HOMO-11 displays an antibonding interaction between Rh1 and S1 as well as Rh2 and S2, that make the Rh1-S1 and Rh2-S2 bonds susceptible for nucleophilic attack. On the other hand, the HOMO-32 reveals conjugated π-interactions in both Co2-Co5-Co7 and Co1-Co5-Co6 planes of the bicapped square pyramidal {Co_6_S} unit (Figure 8). This leads us to presume that these two planes are prone to electrophilic substitution. In addition, HOMO-35 displays orbital overlap between two cobalt atoms and two sulfur atoms of the {Co_2_S_2_} butterfly fragment (Figure S24 in Supplementary Materials).

In order to understand the geometries of many main groups as well as transition metal-fused clusters, cluster electron-counting rules [56,57,58,59] are now well established as conceptual and practical tools. However, both clusters **7** and **8** do not follow any of the cluster-counting rules, including Mingos’ fusion formalism [58] and Jemmis’ mno rule [59]. The electron counts are very complex for both **7** and **8**. This may be due to the fact that these clusters contain multiple fragments and several metal−metal bonds.

## 3. Experimental Section

### 3.1. Materials and Methods

All the manipulations were accomplished in argon atmosphere using schlenk line techniques or inside the glove box. Dichloromethane, Hexane, Toluene, and THF were distilled by using appropriate drying agents (Na/benzophenone) in argon atmosphere prior to use. All chemicals, such as [Co_2_(CO)_8_], [Ru_3_(CO)_12_], [Mo(CO)_6_], [W(CO)_6_], LiBH_4_ (2.0 M in THF) were used as purchased from Sigma Aldrich. The starting materials *nido*-[(Cp*M)_2_B_6_S_2_H_4_(CS_3_)] (Cp* = *ɳ*^5^-C_5_Me_5_, **1**: M = Co, **2**: M = Rh) were synthesized according to the literature procedures [42]. All the syntheses detailed here are reproducible. To separate reaction mixtures, aluminium-supported TLC plates (MERCK) of 250 µm diameter were used. Some 400 and 500 MHz Bruker FT-NMR spectrometers were used to record ^1^H{^11^B} and ^11^B{^1^H} NMR spectra. The residual solvent protons (CDCl_3_, *δ* = 7.26 ppm) were employed as a reference the ^1^H NMR spectra, respectively. The ESI-MS spectra were recorded ln a Bruker MicroTOF-II mass spectrometer. The infrared spectra (IR) were recorded using JASCO FT/IR-1400 spectrometer.

### 3.2. Formation of ***3*** and ***4***

In a flame-dried Schlenk tube under an Ar atmosphere, previously reported *nido*-**1** (0.03 g, 0.048 mmol) was suspended in 5 mL of dry THF at room temperature. A freshly prepared solution of [Mo(CO)_5_·THF] from the photolysis of Mo(CO)_6_ (0.012 g, 0.048 mmol) in THF (5 mL) was added dropwise to the yellow solution of *nido*-**1**. The reaction mixture was irradiated for 3 h, and we observed the color change from yellow to reddish brown. The solvent was removed in the vacuum. Then, the residue was extracted with n-hexane and filtered through 3 cm of celite. The reaction mixture was purified on TLC plates. Elution with a CH_2_Cl_2_/n-hexane (30:70 *v*/*v*) mixture afforded a violet solid **3** (2.9 mg, 7%). In the same reaction conditions, treatment of *nido*-**1** with [W(CO)_5_·THF] prepared from the photolysis of W(CO)_6_ (0.016 g, 0.048 mmol) in THF (5 mL), yielded a red solid **4** (4.14 mg, 9%).

Spectroscopic data of **3**: ^11^B{^1^H} NMR (160 MHz, C_6_D_6_, 22 °C): δ (ppm) = 26.5 (br, 2B), 27.6 (br, 2B), 33.0 (br, 1B), 37.7 (br, 1B); ^1^H NMR (500 MHz, CDCl_3_, 22 °C): δ (ppm) = 1.65 (s, 15H; 1×Cp*), IR (dichloromethane, cm^−1^): ῡ = 2418 (B-H_t_), and 1941 (terminal CO).

Spectroscopic data of **4**: MS (ESI^+^): calcd for [C_26_H_34_B_6_Co_2_O_5_S_5_W]^+^: *m*/*z* 953.9741, found: 953.9784; ^11^B{^1^H} NMR (160 MHz, CDCl_3_, 22 °C): δ (ppm) = 24.6 (br, 2B), 26.4 (br, 1B), 33.4 (br, 2B), 38.2 (br, 1B); ^1^H NMR (500 MHz, CDCl_3_, 22 °C): δ (ppm)= 1.69 (s, 15H; 1×Cp*); ^1^H{^11^B} NMR (500 MHz, CDCl_3_, 22 °C):3.49 (br, 1H, B*H*_t_), 3.75 (s, 2H, B*H*_t_), 4.03 (s, 1H, B*H*_t_); IR (dichloromethane, cm^−1^): ῡ = 2477 (B-H_t_), and 1923 (terminal CO).

### 3.3. Formation of ***5*** and ***6***

In a flame-dried Schlenk tube under an Ar atmosphere, *nido*-**2** (0.02 g, 0.0278 mmol) was suspended in 5 mL of dry THF at room temperature. A freshly prepared solution of [Mo(CO)_5_·THF] from the photolysis of Mo(CO)_6_ (0.007 g, 0.0278 mmol) in THF (5 mL), was added dropwise to the yellow solution of *nido*-**2**. The reaction mixture was irradiated for 3 h, the color changed from yellow to reddish brown. The solvent was removed in the vacuum. Then, the residue was extracted with *n*-hexane and filtered through 3 cm of celite. The reaction mixture was purified on TLC plates. Elution with a CH_2_Cl_2_/*n*-hexane (30:70 *v*/*v*) mixture afforded a orange solid **5** (2.16 mg, 8%). In the same reaction conditions, treatment of *nido*-**2** with [W(CO)_5_·THF] prepared from the photolysis of W(CO)_6_ (0.009 g, 0.0278 mmol) in THF (5 mL), yielded a red solid **6** (2.94 mg, 10%).

Spectroscopic data of **5**: MS (ESI^+^): calcd for [C_26_H_34_B_6_Rh_2_S_5_MoO_5_ – (Mo(CO)_5_) + H]^+^: *m*/*z* 719.0010, found: 719.0036; ^11^B{^1^H} NMR (160 MHz, CDCl_3_, 22 °C): δ (ppm) = 17.3 (br, 2B), 20.3 (br, 1B), 24.6 (br, 2B), 30.4 (br, 1B); ^1^H NMR (500 MHz, CDCl_3_, 22 °C): δ (ppm) = 1.85 (s, 15H; 1×Cp*); ^1^H{^11^B} NMR (500 MHz, CDCl_3_, 22 °C):3.50 (br, 1H, B*H*_t_), 3.75 (s, 2H, B*H*_t_), 4.06 (s, 1H, B*H*_t_); IR (dichloromethane, cm-1): ῡ = 2403 (B-H_t_), 1941 (terminal CO).

Spectroscopic data of **6**: MS (ESI^+^): calcd for [C_26_H_34_B_6_Rh_2_S_5_WO_5_ + NH_4_]^+^: *m*/*z* 1059.9531, found: 1059.9754 ^11^B{^1^H} NMR (160 MHz, C_6_D_6_, 22 °C): δ (ppm) = 16.7 (br, 2B), 20.5 (br, 1B), 23.9 (br, 2B), 24.9 (br, 1B); ^1^H NMR (500 MHz, CDCl_3_, 22 °C): δ (ppm)= 1.80 (s, 15H; 1×Cp*), ^1^H{^11^B} NMR (500 MHz, CDCl_3_, 22 °C): 4.67 (br, 1H, B*H*_t_), 4.75 (s, 1H, B*H*_t_), 5.09 (s, 2H, B*H*_t_); IR (dichloromethane, cm-1): ῡ = 2453 (B-H_t_), 1925 (terminal CO).

### 3.4. Formation of Metallaheteroborane ***7***

In a flame-dried Schlenk tube, under argon atmosphere, a yellow solution of *nido*-1 (0.03 g, 0.048 mmol) in 10 mL dry toluene was charged with [Ru_3_(CO)_12_] (0.061 g, 0.096 mmol) at room temperature. The reaction mixture was stirred for additional 16 h at 80 °C. The solvent was removed under vacuum and the residue was extracted into hexane/CH_2_Cl_2_ (70:30 *v*/*v*) mixture and passed through Celite. Note that compound **7** was isolated in very poor yield and it was combined with the unreacted precursor *nido*-**1** in thin-layered chromatography (TLC). As a result, purification of **7** was not possible for spectroscopic characterization. Although we were not able to purify it, the fractional crystallization allowed us to get few crystals of **7** for X-ray diffraction studies.

### 3.5. Formation of Metallaheteroborane ***8***

Under argon atmosphere, in a flame-dried Schlenk tube, the yellow solution of *nido*-**2** (0.02 g, 0.0278 mmol) in dry toluene (10 mL) was charged dropwise with a solution of [Co_2_(CO)_8_] (0.019 g, 0.0556 mmol) in 5 mL dry toluene at room temperature for 16 h. The colour of the reaction mixture was changed from yellow to deep brown. The solvent was removed under vacuum and the residue was extracted into hexane/CH_2_Cl_2_ (70:30 *v*/*v*) mixture and passed through celite. Compound **8** was isolated in very poor yield that was merged with unreacted precursor *nido*-**2** in thin-layered chromatography (TLC). Thus, the purification of **8** for spectroscopic data was not possible. Although we were not able to purify it, fractional crystallization allowed us to get few crystals of **8** for X-ray diffraction studies.

### 3.6. X-ray Structure Determination

Suitable crystals of **4**, **5**, **6**, **7** and **8** were grown at −5 °C by slow diffusion of a CH_2_Cl_2_-hexane solution. The X-ray data were collected and integrated by using D8 VENTURE Bruker AXS for **4**, **5**, **6** and **7** with a CMOS-PHOTON70 detector having multilayer device monochromated Mo-Kα (λ = 0.71073 Å) radiation at 150(2) K. For **8**, the X-ray data were collected and integrated by using Bruker D8 VENTURE diffractometer with PHOTON II detector having graphite monochromated Mo-Kα (λ = 0.71073 Å) radiation at 297(2) K. Using SHELXT-2014, SHELXS-97, [60,61] the structures were solved and using SHELXL-2014, SHELXL-2017, SHELXL-2018, and in [62] the structures were refined. For 5, The contribution of the disordered solvents to the calculated structure factors was estimated following the BYPASS algorithm [63], implemented as the SQUEEZE option in PLATON [64]. A new data set, free of solvent contribution, was then used in the final refinement. All non-Hydrogen atoms were refined with anisotropic atomic displacement parameters. Except Hydrogen atoms linked to Boron atoms that were introduced in the structural model through Fourier difference maps analysis, H atoms were finally included in their calculated positions and treated as riding on their parent atom with constrained thermal parameters. For 8, due to the highly disordered nature of cyclopentadienyl moieties, SHELXL restraint, RIGU, had to be used to impose physically reasonable relative motion of the atoms. It restrains the anisotropic displacement parameters of bonded atoms to be similar along the bond. A PLATON/check cif indicated that there are solvent accessible voids in the lattice. However, the solvent (CH_2_Cl_2_) could not be modelled from difference electron density peaks. Hence, it was decided to squeeze the electron densities corresponding to the disordered solvent molecules. PLATON/SQUEEZE [65] (Version = 10719) program was used to find out solvent accessible volume and electron counts. A total number of 73 electrons were found in the void with a solvent accessible volume of 439 Å^3,^ which corresponds to 16% of the unit cell volume. Without solvent molecules R1 = 0.033 for 10153 reflections of Fo > 4sig(Fo) and wR2 0.1078 for all data. With solvent contribution (SQUEEZE) R1 = 0.029 for 10145 reflections of Fo > 4sig(Fo) and wR2 0.076 for all data. All the structures of the clusters were drawn using Olex2 [66]. The Cambridge Crystallographic Data Center has been provided with the crystallographic data of the molecules as supplementary publications no CCDC- 2211791 (**4**), 2211792 (**5**), 2214567 (**6**), 2214528 (**7**), and 2144409 (**8**) contain crystallographic data. These data can be obtained free of charge from the Cambridge Crystallographic Data Centre via www.ccdc.cam.ac.uk/data_request/cif (accessed on 7 October 2022). 

Crystal data of **4**. C_26_H_34_B_6_Co_2_O_5_S_5_W, formula weight, *M_r_*.= 953.40, Monoclinic, *P 2_1_/c*, unit cell, *a* = 15.603(2) Å, *b* = 17.630(2) Å, *c* = 15.3019(19) Å, *α* = 90°, *β* = 118.175(4)°, *γ* = 90°; *Z* = 4; *V* = 3710.5(8) Å^3^; *μ* = 4.291 mm^–1^; *F*(000) = 1872; *ρ_calcd_* = 1.707 g/cm^3^; *R_1_* = 0.0735; w*R_2_* = 0.1947; 8479 independent reflections [2θ ≤ 55.04°], and 417 parameters.

Crystal data of **5**. C_26_H_34_B_6_MoO_5_Rh_2_S_5_, formula weight, *M_r_*.= 953.45, orthorhombic, *Pbcn*, unit cell, *a* = 18.0346(10) Å, *b* = 15.4968(12) Å, *c* = 27.550(2) Å, *α* = 90°, *β* = 90°, *γ* = 90°; *Z* = 8; *V* = 7699.6(9) Å^3^; *μ* = 1.471 mm^–1^; *F*(000) = 3776; *ρ_calcd_* = 1.645 g/cm^3^; *R_1_* = 0.0422; w*R_2_* = 0.0969; 8750 independent reflections [2θ ≤ 54.99°], and 428 parameters.

Crystal data of **6**. C_26_H_34_B_6_O_5_Rh_2_S_5_W, formula weight, *M_r_*.= 1041.36, orthorhombic, *Pbcn*, unit cell, *a* = 18.0153(15) Å, *b* = 15.5395(16) Å, *c* = 27.555(3) Å, *α* = 90°, *β* = 90°, *γ* = 90°; *Z* = 8; *V* = 7714.0(13) Å^3^; *μ* = 4.124 mm^–1^; *F*(000) = 4032; *ρ_calcd_* = 1.793 g/cm^3^; *R_1_* = 0.0219; w*R_2_* = 0.0494; 8770 independent reflections [2θ ≤ 54.99°], and 428 parameters.

Crystal data of **7**. C_39_H_36_B_6_Co_2_O_18_Ru_7_S_5_, 2(CH_2_Cl_2_); formula weight, *M_r_*.= 2013.04, triclinic, *P-1*, unit cell, *a* = 11.6527(14) Å, *b* = 13.0484(12) Å, *c* = 21.925(2) Å, *α* = 94.971(4)°, *β* = 105.005(5)°, *γ* = 99.109(5)°; *Z* = 2; *V* = 3150.8(6) Å^3^; *μ* = 2.539 mm^–1^; *F*(000) = 1940; *ρ_calcd_* = 2.122 g/cm^3^; *R_1_* = 0.0559; w*R_2_* = 0.1293; 14194 independent reflections [2θ ≤ 54.97°], and 758 parameters.

Crystal data of **8**. C_33_H_34_B_6_Co_6_O_13_Rh_2_S_5_, formula weight, *M_r_*.= 1423.16, Triclinic, *P-1*, unit cell, *a* = 12.4725(4) Å, *b* = 14.4351(5) Å, *c* = 16.0368(5) Å, *α* = 88.7070(10)°, *β* = 74.7360(10)°, *γ* = 79.9110°; *Z* = 2; *V* = 2741.52(16) Å^3^; *μ* = 2.594 mm^–1^; *F*(000) = 1396; *ρ_calcd_* = 1.724 g/cm^3^; *R_1_* = 0.0295; w*R_2_* = 0.0764; 13201 independent reflections [2θ ≤ 56.00°], and 596 parameters.

### 3.7. Computational Details

All molecules were fully optimized using the BP86 functional [67,68], in conjunction with a def2-svp basis set using the Gaussian 16 program (Gaussian, Wallingford, CT, USA) [69]. All compounds were fully optimized in gaseous state using their X-ray crystallographic structures. The calculations were performed with the Cp analogues, instead of Cp*, to save computing time. All the optimized structures and orbital graphics were produced using Gaussview [70] and Chemcraft [71].

## 4. Conclusions

In summary, we have synthesized and structurally characterized numerous exciting clusters **3**–**6** in which the S atom of the {B_2_CS_3_} moiety is coordinated to group 6 metal carbonyl fragments in *η*^1^-fashion. On the other hand, we have established the structures of clusters **7** and **8** having very characteristic and unusual fusion. The molecular structure of cluster **7** consists of a butterfly {Ru_3_S} moiety that is almost flat. Cluster **8** shows bicapped square pyramidal {Co_6_S} unit that is fused with a butterfly {Co_2_S_2_} via a common {Co_2_} edge. Although both the clusters are of condensed types, the electron counts are very complex and they do not follow any of the cluster-counting rules, including Mingos’ fusion formalism and Jemmis’ mno rule.

## Data Availability

Supporting data reported can be found as Supplementary Materials.

## Supplementary Materials

The following supporting information can be downloaded at: https://www.mdpi.com/article/10.3390/molecules28030998/s1, Figures S1–S4: Molecular structures and labelling diagrams of of **4**–**8**; Figures S5–S19: spectroscopic details of **3**–**6**, Figures S20–S21: selected molecular orbitals of **7** and **8**, Figures S22–S27: Optimized geometries of **4**–**8** [72,73,74].

## Sample Availability

Samples of the compounds are available from the authors upon request.
