# Supplementary material for: Chemistry of CS2 and CS3 Bridged Decaborane Analogues: Regular Coordination Versus Cluster Expansion
Source: Molecules. 2023 Jan 19;28(3):998. doi: 10.3390/molecules28030998 (PMC9919328; doi:10.3390/molecules28030998)

checkCIF/PLATON report

Structure factors have been supplied for datablock(s) SGGAR2

THIS REPORT IS FOR GUIDANCE ONLY. IF USED AS PART OF A REVIEW PROCEDURE FOR PUBLICATION, IT SHOULD NOT REPLACE THE EXPERTISE OF AN EXPERIENCED CRYSTALLOGRAPHIC REFEREE.

No syntax errors found. CIF dictionary Interpreting this report

Datablock: SGGAR2

Bond precision: C-C = 0.0083 A Wavelength=0.71073

Cell:	a=18.0346(10)	b=15.4968(12)	c=27.550(2)
	alpha=90	beta=90	gamma=90
Temperature:	150 K		

	Calculated	Reported
Volume	7699.6(9)	7699.6(9)
Space group	P b c n	P b c n
Hall group	-P 2n 2ab	-P 2n 2ab
Moiety formula	C26.20 H34.40 B6 Cl0.40 Mo O5 Rh2 S5, 0.8(C H2 Cl2)	C27 H36 B6 Cl2 Mo O5 Rh2 S5
Sum formula	C27 H36 B6 Cl2 Mo O5 Rh2 S5	C27 H36 B6 Cl2 Mo O5 Rh2 S5
Mr	1038.38	1038.38
Dx,g cm-3	1.792	1.792
Z	8	8
Mu (mm-1)	1.614	1.614
F000	4112.0	4112.0
F000'	4088.63	
h,k,lmax	23,20,35	22,20,35
Nref	8840	8745
Tmin,Tmax	0.755,0.937	0.807,0.937
Tmin'	0.740	

Correction method= # Reported T Limits: Tmin=0.807 Tmax=0.937
AbsCorr = MULTI-SCAN

Data completeness= 0.989 Theta(max)= 27.493

R(reflections)= 0.0488(6645) wR2(reflections)= 0.1340(8745)

S = 1.050 Npar= 464

The following ALERTS were generated. Each ALERT has the format
test-name_ALERT_alert-type_alert-level.
Click on the hyperlinks for more details of the test.

Alert level B

```
PLAT220_ALERT_2_B NonSolvent   Resd 1   C   Ueq(max)/Ueq(min) Range        7.4 Ratio
```

Alert level C

```
PLAT222_ALERT_3_C NonSolvent Resd 1  H   Uiso(max)/Uiso(min) Range        7.1 Ratio
PLAT242_ALERT_2_C Low    'MainMol' Ueq as Compared to Neighbors of        Mo1 Check
PLAT260_ALERT_2_C Large Average Ueq of Residue Including       Cl1      0.153 Check
PLAT260_ALERT_2_C Large Average Ueq of Residue Including      Cl1C      0.153 Check
PLAT303_ALERT_2_C Full Occupancy Atom H19B      with # Connections       1.20 Check
PLAT342_ALERT_3_C Low Bond Precision on  C-C Bonds ...............     0.0083 Ang.
PLAT906_ALERT_3_C Large K Value in the Analysis of Variance ......      2.450 Check
PLAT911_ALERT_3_C Missing FCF Refl Between Thmin & STh/L=    0.600          9 Report
PLAT971_ALERT_2_C Check Calcd Resid. Dens.  0.45A   From Mo1             2.12 eA-3
PLAT971_ALERT_2_C Check Calcd Resid. Dens.  0.30A   From Mo1             2.00 eA-3
PLAT971_ALERT_2_C Check Calcd Resid. Dens.  0.36A   From Mo1             1.96 eA-3
PLAT971_ALERT_2_C Check Calcd Resid. Dens.  0.33A   From Cl1C            1.84 eA-3
PLAT972_ALERT_2_C Check Calcd Resid. Dens.  0.56A   From Cl2C           -1.67 eA-3
PLAT972_ALERT_2_C Check Calcd Resid. Dens.  0.53A   From Cl2C           -1.67 eA-3
PLAT972_ALERT_2_C Check Calcd Resid. Dens.  0.67A   From Cl1C           -1.57 eA-3
PLAT972_ALERT_2_C Check Calcd Resid. Dens.  0.07A   From Cl1B           -1.54 eA-3
```

Alert level G

```
PLAT002_ALERT_2_G Number of Distance or Angle Restraints on AtSite          9 Note
PLAT003_ALERT_2_G Number of Uiso or Uij Restrained non-H Atoms ...          9 Report
PLAT004_ALERT_5_G Polymeric Structure Found with Maximum Dimension          2 Info
PLAT042_ALERT_1_G Calc. and Reported Moiety Formula Strings Differ     Please Check
PLAT083_ALERT_2_G SHELXL Second Parameter in WGHT  Unusually Large      25.51 Why ?
PLAT171_ALERT_4_G The CIF-Embedded .res File Contains EADP Records          1 Report
PLAT172_ALERT_4_G The CIF-Embedded .res File Contains DFIX Records          2 Report
PLAT186_ALERT_4_G The CIF-Embedded .res File Contains ISOR Records          1 Report
PLAT230_ALERT_2_G Hirshfeld Test Diff for    Cl1B     --O3_b      .      15.5 s.u.
PLAT232_ALERT_2_G Hirshfeld Test Diff (M-X)  Mo1      --S5        .      11.6 s.u.
PLAT232_ALERT_2_G Hirshfeld Test Diff (M-X)  Mo1      --C22       .       6.7 s.u.
PLAT232_ALERT_2_G Hirshfeld Test Diff (M-X)  Mo1      --C23       .       7.2 s.u.
PLAT232_ALERT_2_G Hirshfeld Test Diff (M-X)  Mo1      --C24       .       7.0 s.u.
PLAT301_ALERT_3_G Main Residue  Disorder ..............(Resd  1  )         1% Note
PLAT302_ALERT_4_G Anion/Solvent/Minor-Residue Disorder (Resd  2  )       100% Note
PLAT302_ALERT_4_G Anion/Solvent/Minor-Residue Disorder (Resd  3  )       100% Note
PLAT367_ALERT_2_G Long?  C(sp?)-C(sp?) Bond  C14      - C19       .      1.50 Ang.
PLAT432_ALERT_2_G Short Inter X...Y Contact  O4       ..C27C             2.15 Ang.
                                       -x,1-y,-z  =      5_565 Check
PLAT432_ALERT_2_G Short Inter X...Y Contact  C25      ..C27C             2.40 Ang.
                                       -x,1-y,-z  =      5_565 Check
PLAT780_ALERT_1_G Coordinates do not Form a Properly Connected Set     Please Do !
PLAT789_ALERT_4_G Atoms with Negative _atom_site_disorder_group  #         15 Check
PLAT860_ALERT_3_G Number of Least-Squares Restraints .............         64 Note
PLAT883_ALERT_1_G No Info/Value for _atom_sites_solution_primary .     Please Do !
PLAT910_ALERT_3_G Missing # of FCF Reflection(s) Below Theta(Min).          4 Note
```

```
PLAT912_ALERT_4_G Missing # of FCF Reflections Above STh/L=  0.600         63 Note
PLAT933_ALERT_2_G Number of OMIT Records in Embedded .res File ...          6 Note
PLAT941_ALERT_3_G Average HKL Measurement Multiplicity ..........         4.9 Low
PLAT978_ALERT_2_G Number C-C Bonds with Positive Residual Density.          1 Info
```

```
   0 ALERT level A = Most likely a serious problem - resolve or explain
   1 ALERT level B = A potentially serious problem, consider carefully
  16 ALERT level C = Check. Ensure it is not caused by an omission or oversight
  28 ALERT level G = General information/check it is not something unexpected

   3 ALERT type 1 CIF construction/syntax error, inconsistent or missing data
  26 ALERT type 2 Indicator that the structure model may be wrong or deficient
   8 ALERT type 3 Indicator that the structure quality may be low
   7 ALERT type 4 Improvement, methodology, query or suggestion
   1 ALERT type 5 Informative message, check
```

It is advisable to attempt to resolve as many as possible of the alerts in all categories. Often the minor alerts point to easily fixed oversights, errors and omissions in your CIF or refinement strategy, so attention to these fine details can be worthwhile. In order to resolve some of the more serious problems it may be necessary to carry out additional measurements or structure refinements. However, the purpose of your study may justify the reported deviations and the more serious of these should normally be commented upon in the discussion or experimental section of a paper or in the "special_details" fields of the CIF. checkCIF was carefully designed to identify outliers and unusual parameters, but every test has its limitations and alerts that are not important in a particular case may appear. Conversely, the absence of alerts does not guarantee there are no aspects of the results needing attention. It is up to the individual to critically assess their own results and, if necessary, seek expert advice.

Publication of your CIF in IUCr journals

A basic structural check has been run on your CIF. These basic checks will be run on all CIFs submitted for publication in IUCr journals (*Acta Crystallographica*, *Journal of Applied Crystallography*, *Journal of Synchrotron Radiation*); however, if you intend to submit to *Acta Crystallographica Section C* or *E* or *IUCrData*, you should make sure that full publication checks are run on the final version of your CIF prior to submission.

Publication of your CIF in other journals

Please refer to the *Notes for Authors* of the relevant journal for any special instructions relating to CIF submission.

PLATON version of 13/07/2021; check.def file version of 13/07/2021

Datablock SGGAR2 - ellipsoid plot

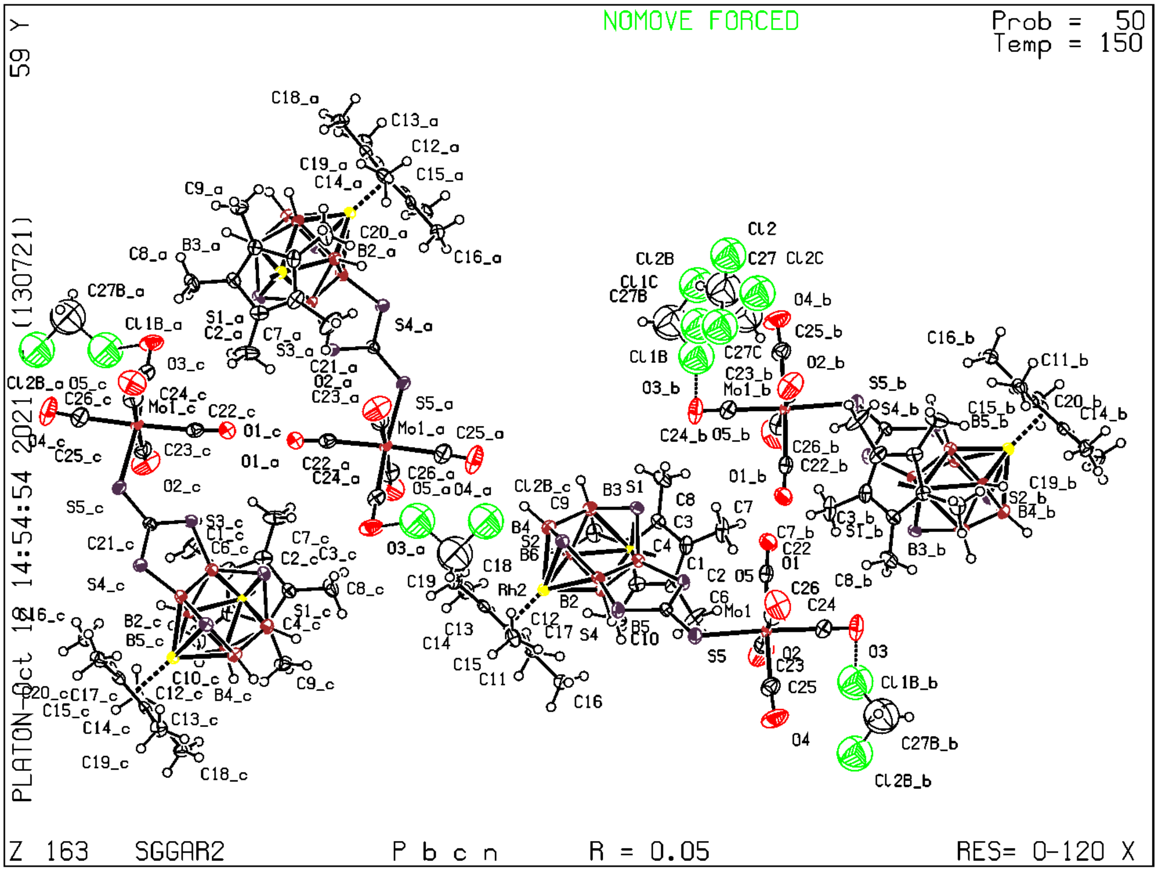

Supplement: Supplementary file 1 [file molecules-28-00998-s001.zip › CheckCIF/checkcif of 5.pdf]
