# Supplementary material for: Chemistry of CS2 and CS3 Bridged Decaborane Analogues: Regular Coordination Versus Cluster Expansion
Source: Molecules. 2023 Jan 19;28(3):998. doi: 10.3390/molecules28030998 (PMC9919328; doi:10.3390/molecules28030998)

checkCIF/PLATON report

Structure factors have been supplied for datablock(s) SGGAR5

THIS REPORT IS FOR GUIDANCE ONLY. IF USED AS PART OF A REVIEW PROCEDURE FOR PUBLICATION, IT SHOULD NOT REPLACE THE EXPERTISE OF AN EXPERIENCED CRYSTALLOGRAPHIC REFEREE.

No syntax errors found. CIF dictionary Interpreting this report

Datablock: SGGAR5

```
Bond precision:   C-C = 0.0040 A          Wavelength=0.71073

Cell:             a=18.0153(15)      b=15.5395(16)      c=27.555(3)
                  alpha=90           beta=90            gamma=90
Temperature:      150 K
```

	Calculated	Reported
Volume	7714.0(13)	7714.0(13)
Space group	P b c n	P b c n
Hall group	-P 2n 2ab	-P 2n 2ab
Moiety formula	C26 H34 B6 O5 Rh2 S5 W [+ solvent]	C26 H34 B6 O5 Rh2 S5 W
Sum formula	C26 H34 B6 O5 Rh2 S5 W [+ solvent]	C26 H34 B6 O5 Rh2 S5 W
Mr	1041.35	1041.36
Dx,g cm-3	1.793	1.793
Z	8	8
Mu (mm-1)	4.124	4.124
F000	4032.0	4032.0
F000’	4013.48	
h,k,lmax	23,20,35	23,19,35
Nref	8858	8770
Tmin,Tmax	0.522,0.814	0.680,0.814
Tmin’	0.512	

```
Correction method= # Reported T Limits: Tmin=0.680 Tmax=0.814
AbsCorr = MULTI-SCAN

Data completeness= 0.990           Theta(max)= 27.496
```

R(reflections)= 0.0219(7429) wR2(reflections)= 0.0494(8770)

S = 0.954 Npar= 428

The following ALERTS were generated. Each ALERT has the format
test-name_ALERT_alert-type_alert-level.
Click on the hyperlinks for more details of the test.

Alert level C

```
PLAT911_ALERT_3_C Missing FCF Refl Between Thmin & STh/L=    0.600         11 Report
```

Alert level G

```
PLAT232_ALERT_2_G Hirshfeld Test Diff (M-X)  W1       --C31      .        6.5 s.u.
PLAT232_ALERT_2_G Hirshfeld Test Diff (M-X)  W1       --C35      .        8.0 s.u.
PLAT232_ALERT_2_G Hirshfeld Test Diff (M-X)  W1       --C37      .        6.5 s.u.
PLAT232_ALERT_2_G Hirshfeld Test Diff (M-X)  W1       --C39      .        6.2 s.u.
PLAT380_ALERT_4_G Incorrectly? Oriented X(sp2)-Methyl Moiety .....        C10 Check
PLAT380_ALERT_4_G Incorrectly? Oriented X(sp2)-Methyl Moiety .....        C18 Check
PLAT605_ALERT_4_G Largest Solvent Accessible VOID in the Structure        138 A**3
PLAT869_ALERT_4_G ALERTS Related to the Use of SQUEEZE  Suppressed          ! Info
PLAT910_ALERT_3_G Missing # of FCF Reflection(s) Below Theta(Min).          2 Note
PLAT912_ALERT_4_G Missing # of FCF Reflections Above STh/L=  0.600         63 Note
PLAT913_ALERT_3_G Missing # of Very Strong Reflections in FCF ....          1 Note
PLAT933_ALERT_2_G Number of HKL-OMIT Records in Embedded .res File          3 Note
PLAT941_ALERT_3_G Average HKL Measurement Multiplicity ..........         1.0 Low
PLAT978_ALERT_2_G Number C-C Bonds with Positive Residual Density.          6 Info
```

---

0 **ALERT level A** = Most likely a serious problem - resolve or explain
0 **ALERT level B** = A potentially serious problem, consider carefully
1 **ALERT level C** = Check. Ensure it is not caused by an omission or oversight
14 **ALERT level G** = General information/check it is not something unexpected

0 ALERT type 1 CIF construction/syntax error, inconsistent or missing data
6 ALERT type 2 Indicator that the structure model may be wrong or deficient
4 ALERT type 3 Indicator that the structure quality may be low
5 ALERT type 4 Improvement, methodology, query or suggestion
0 ALERT type 5 Informative message, check

It is advisable to attempt to resolve as many as possible of the alerts in all categories. Often the minor alerts point to easily fixed oversights, errors and omissions in your CIF or refinement strategy, so attention to these fine details can be worthwhile. In order to resolve some of the more serious problems it may be necessary to carry out additional measurements or structure refinements. However, the purpose of your study may justify the reported deviations and the more serious of these should normally be commented upon in the discussion or experimental section of a paper or in the "special_details" fields of the CIF. checkCIF was carefully designed to identify outliers and unusual parameters, but every test has its limitations and alerts that are not important in a particular case may appear. Conversely, the absence of alerts does not guarantee there are no aspects of the results needing attention. It is up to the individual to critically assess their own results and, if necessary, seek expert advice.

Publication of your CIF in IUCr journals

A basic structural check has been run on your CIF. These basic checks will be run on all CIFs submitted for publication in IUCr journals (*Acta Crystallographica*, *Journal of Applied Crystallography*, *Journal of Synchrotron Radiation*); however, if you intend to submit to *Acta Crystallographica Section C* or *E* or *IUCrData*, you should make sure that full publication checks are run on the final version of your CIF prior to submission.

Publication of your CIF in other journals

Please refer to the *Notes for Authors* of the relevant journal for any special instructions relating to CIF submission.

PLATON version of 28/11/2022; check.def file version of 28/11/2022

Datablock SGGAR5 - ellipsoid plot

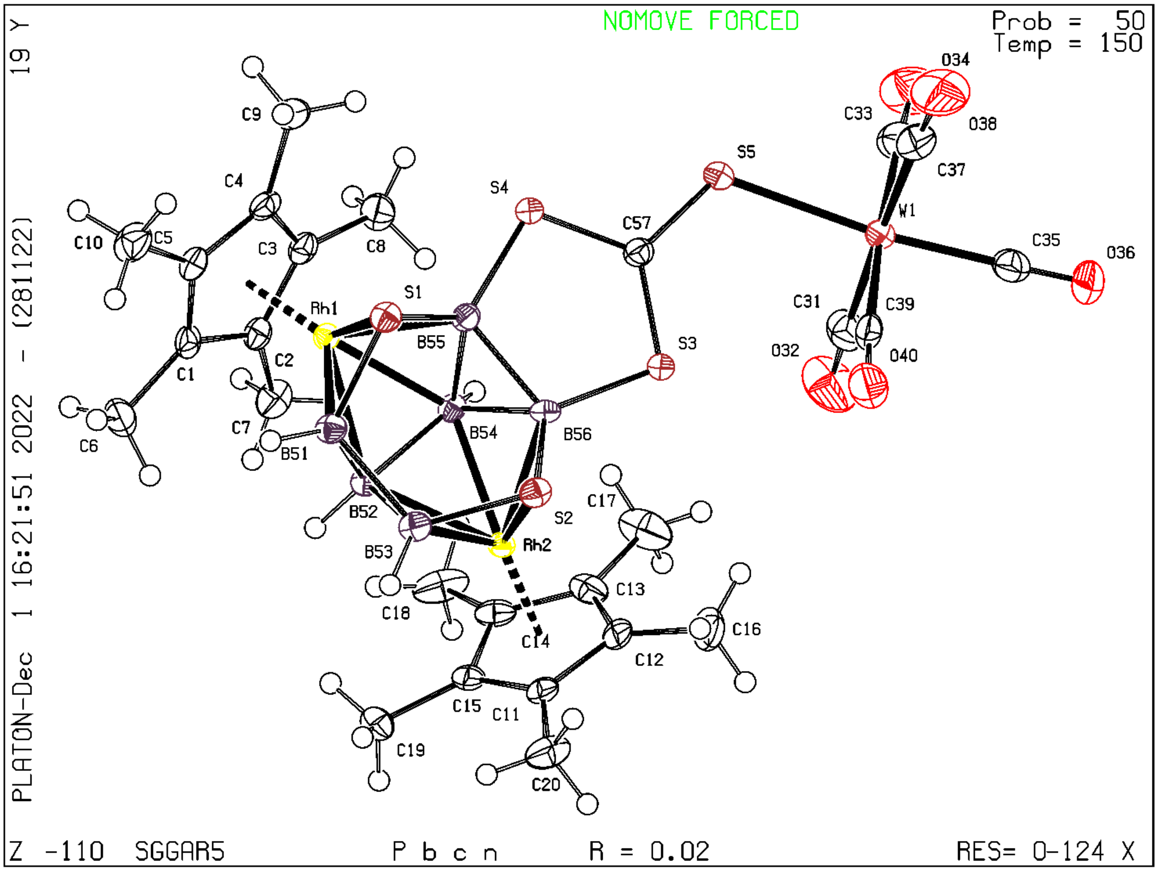

Supplement: Supplementary file 1 [file molecules-28-00998-s001.zip › CheckCIF/checkcif of 6.pdf]
