# Supplementary material for: Chemistry of CS2 and CS3 Bridged Decaborane Analogues: Regular Coordination Versus Cluster Expansion
Source: Molecules. 2023 Jan 19;28(3):998. doi: 10.3390/molecules28030998 (PMC9919328; doi:10.3390/molecules28030998)

checkCIF/PLATON report

Structure factors have been supplied for datablock(s) SGGAR4

THIS REPORT IS FOR GUIDANCE ONLY. IF USED AS PART OF A REVIEW PROCEDURE FOR PUBLICATION, IT SHOULD NOT REPLACE THE EXPERTISE OF AN EXPERIENCED CRYSTALLOGRAPHIC REFEREE.

No syntax errors found. CIF dictionary Interpreting this report

Datablock: SGGAR4

Bond precision: C-C = 0.0126 A Wavelength=0.71073

Cell:	a=11.6527(14)	b=13.0484(12)	c=21.925(2)
	alpha=94.971(4)	beta=105.005(5)	gamma=99.109(5)
Temperature:	150 K		

	Calculated	Reported
Volume	3150.8(6)	3150.8(6)
Space group	P -1	P -1
Hall group	-P 1	-P 1
Moiety formula	C39 H36 B6 Co2 O18 Ru7 S5, 2(C H2 Cl2)	C39 H36 B6 Co2 O18 Ru7 S5, 2(C H2 Cl2)
Sum formula	C41 H40 B6 Cl4 Co2 O18 Ru7 S5	C41 H40 B6 Cl4 Co2 O18 Ru7 S5
Mr	2013.04	2013.04
Dx,g cm-3	2.122	2.122
Z	2	2
Mu (mm-1)	2.539	2.539
F000	1940.0	1940.0
F000'	1926.74	
h,k,lmax	15,16,28	15,16,28
Nref	14436	14194
Tmin,Tmax	0.760,0.938	0.682,0.938
Tmin'	0.353	

Correction method= # Reported T Limits: Tmin=0.682 Tmax=0.938
AbsCorr = MULTI-SCAN

Data completeness= 0.983 Theta(max)= 27.487

R(reflections)= 0.0559(12099) wR2(reflections)= 0.1293(14194)

S = 1.104 Npar= 758

The following ALERTS were generated. Each ALERT has the format
test-name_ALERT_alert-type_alert-level.
Click on the hyperlinks for more details of the test.

Alert level C

```
PLAT220_ALERT_2_C NonSolvent   Resd 1  C   Ueq(max)/Ueq(min) Range        3.4 Ratio
PLAT222_ALERT_3_C NonSolvent Resd 1  H   Uiso(max)/Uiso(min) Range        5.3 Ratio
PLAT244_ALERT_4_C Low    'Solvent' Ueq as Compared to Neighbors of       C111 Check
PLAT244_ALERT_4_C Low    'Solvent' Ueq as Compared to Neighbors of       C121 Check
PLAT260_ALERT_2_C Large Average Ueq of Residue Including      Cl11      0.107 Check
PLAT260_ALERT_2_C Large Average Ueq of Residue Including      Cl21      0.127 Check
PLAT342_ALERT_3_C Low Bond Precision on  C-C Bonds ..............     0.0126 Ang.
PLAT906_ALERT_3_C Large K Value in the Analysis of Variance ......      4.388 Check
PLAT910_ALERT_3_C Missing # of FCF Reflection(s) Below Theta(Min).          9 Note
PLAT911_ALERT_3_C Missing FCF Refl Between Thmin & STh/L=    0.600         56 Report
PLAT972_ALERT_2_C Check Calcd Resid. Dens.  0.52Ang From Cl12           -2.28 eA-3
PLAT972_ALERT_2_C Check Calcd Resid. Dens.  0.84Ang From Cl12           -2.11 eA-3
PLAT977_ALERT_2_C Check Negative Difference Density on H11A      .      -0.35 eA-3
PLAT977_ALERT_2_C Check Negative Difference Density on H11B      .      -0.54 eA-3
PLAT977_ALERT_2_C Check Negative Difference Density on H76A      .      -0.38 eA-3
PLAT977_ALERT_2_C Check Negative Difference Density on H80B      .      -0.33 eA-3
PLAT977_ALERT_2_C Check Negative Difference Density on H91       .      -0.80 eA-3
PLAT977_ALERT_2_C Check Negative Difference Density on H92       .      -0.98 eA-3
```

Alert level G

```
PLAT083_ALERT_2_G SHELXL Second Parameter in WGHT  Unusually Large      43.02 Why ?
PLAT303_ALERT_2_G Full Occupancy Atom H91       with # Connections       3.00 Check
PLAT912_ALERT_4_G Missing # of FCF Reflections Above STh/L=  0.600        180 Note
PLAT913_ALERT_3_G Missing # of Very Strong Reflections in FCF ....          3 Note
PLAT941_ALERT_3_G Average HKL Measurement Multiplicity ..........         1.0 Low
PLAT978_ALERT_2_G Number C-C Bonds with Positive Residual Density.          0 Info
```

---

0 **ALERT level A** = Most likely a serious problem - resolve or explain
0 **ALERT level B** = A potentially serious problem, consider carefully
18 **ALERT level C** = Check. Ensure it is not caused by an omission or oversight
6 **ALERT level G** = General information/check it is not something unexpected

0 ALERT type 1 CIF construction/syntax error, inconsistent or missing data
14 ALERT type 2 Indicator that the structure model may be wrong or deficient
7 ALERT type 3 Indicator that the structure quality may be low
3 ALERT type 4 Improvement, methodology, query or suggestion
0 ALERT type 5 Informative message, check

It is advisable to attempt to resolve as many as possible of the alerts in all categories. Often the minor alerts point to easily fixed oversights, errors and omissions in your CIF or refinement strategy, so attention to these fine details can be worthwhile. In order to resolve some of the more serious problems it may be necessary to carry out additional measurements or structure refinements. However, the purpose of your study may justify the reported deviations and the more serious of these should normally be commented upon in the discussion or experimental section of a paper or in the "special_details" fields of the CIF. checkCIF was carefully designed to identify outliers and unusual parameters, but every test has its limitations and alerts that are not important in a particular case may appear. Conversely, the absence of alerts does not guarantee there are no aspects of the results needing attention. It is up to the individual to critically assess their own results and, if necessary, seek expert advice.

Publication of your CIF in IUCr journals

A basic structural check has been run on your CIF. These basic checks will be run on all CIFs submitted for publication in IUCr journals (*Acta Crystallographica*, *Journal of Applied Crystallography*, *Journal of Synchrotron Radiation*); however, if you intend to submit to *Acta Crystallographica Section C* or *E* or *IUCrData*, you should make sure that full publication checks are run on the final version of your CIF prior to submission.

Publication of your CIF in other journals

Please refer to the *Notes for Authors* of the relevant journal for any special instructions relating to CIF submission.

PLATON version of 28/11/2022; check.def file version of 28/11/2022

Datablock SGGAR4 - ellipsoid plot

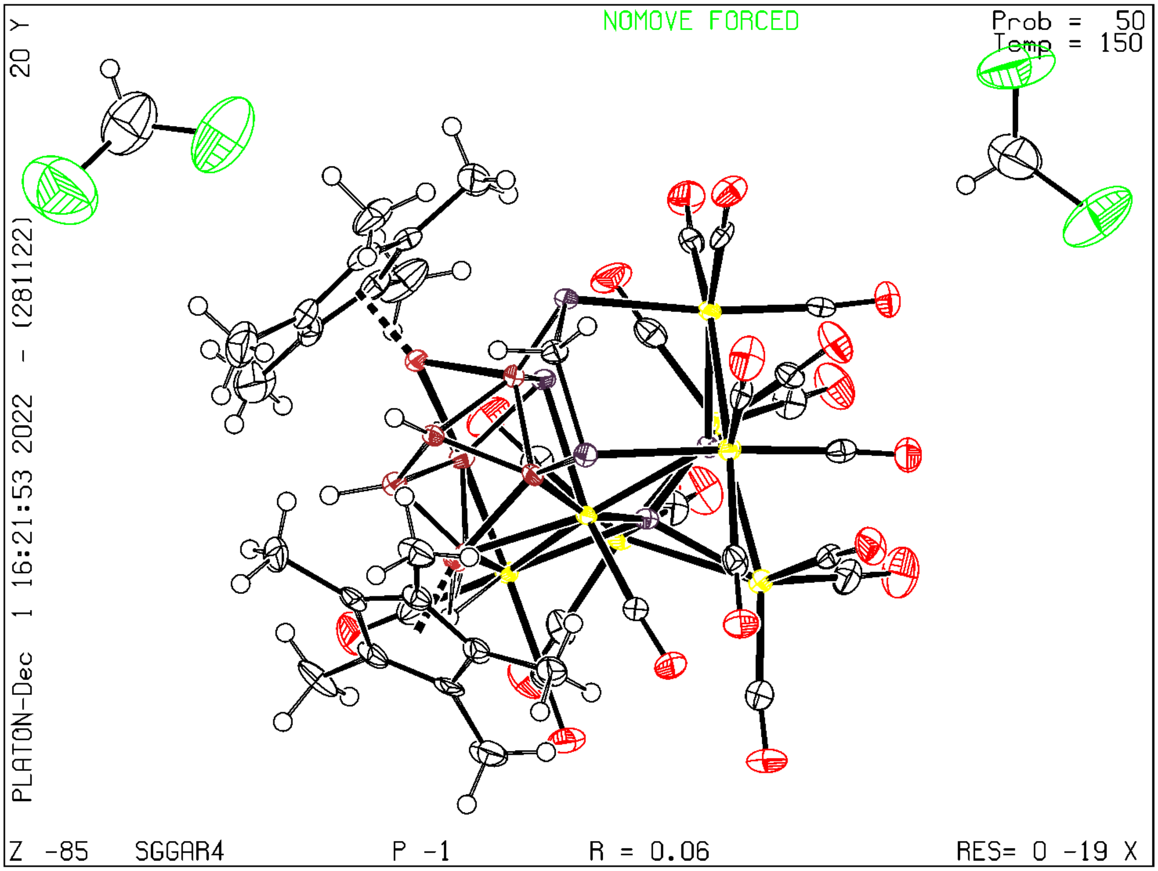

Supplement: Supplementary file 1 [file molecules-28-00998-s001.zip › CheckCIF/checkcif of 7.pdf]
