# Supplementary material for: Chemistry of CS2 and CS3 Bridged Decaborane Analogues: Regular Coordination Versus Cluster Expansion
Source: Molecules. 2023 Jan 19;28(3):998. doi: 10.3390/molecules28030998 (PMC9919328; doi:10.3390/molecules28030998)

checkCIF/PLATON report

Structure factors have been supplied for datablock(s) shelx

THIS REPORT IS FOR GUIDANCE ONLY. IF USED AS PART OF A REVIEW PROCEDURE FOR PUBLICATION, IT SHOULD NOT REPLACE THE EXPERTISE OF AN EXPERIENCED CRYSTALLOGRAPHIC REFEREE.

No syntax errors found. CIF dictionary Interpreting this report

Datablock: shelx

Bond precision: C-C = 0.0070 A Wavelength=0.71073

Cell:	a=12.4725(4)	b=14.4351(5)	c=16.0368(5)
	alpha=88.707(1)	beta=74.736(1)	gamma=79.911(1)
Temperature:	297 K		

	Calculated	Reported
Volume	2741.52(16)	2741.52(16)
Space group	P -1	P -1
Hall group	-P 1	-P 1
Moiety formula	C33 H34 B6 Co6 O13 Rh2 S5 [+ solvent]	C33 H34 B6 Co6 O13 Rh2 S5 [+ solvent]
Sum formula	C33 H34 B6 Co6 O13 Rh2 S5 [+ solvent]	C33 H34 B6 Co6 O13 Rh2 S5
Mr	1423.16	1423.16
Dx,g cm-3	1.724	1.724
Z	2	2
Mu (mm-1)	2.594	2.594
F000	1396.0	1396.0
F000’	1397.35	
h,k,lmax	16,19,21	16,19,21
Nref	13235	13201
Tmin,Tmax	0.740,0.949	0.622,0.747
Tmin’	0.725	

Correction method= # Reported T Limits: Tmin=0.622 Tmax=0.747
AbsCorr = MULTI-SCAN

Data completeness= 0.997 Theta(max)= 28.000

R(reflections)= 0.0295(10147) wR2(reflections)= 0.0764(13201)

S = 1.049 Npar= 596

The following ALERTS were generated. Each ALERT has the format
test-name_ALERT_alert-type_alert-level.
Click on the hyperlinks for more details of the test.

Alert level B

```
PLAT910_ALERT_3_B Missing # of FCF Reflection(s) Below Theta(Min).          25 Note
```

Author Response: These are outlier reflections which are omitted from refinement

---

Alert level C

```
PLAT213_ALERT_2_C Atom C6               has ADP max/min Ratio .....          3.3 prolat
PLAT213_ALERT_2_C Atom C8               has ADP max/min Ratio .....          3.6 prolat
PLAT213_ALERT_2_C Atom C9               has ADP max/min Ratio .....          3.7 prolat
PLAT213_ALERT_2_C Atom C10              has ADP max/min Ratio .....          3.5 prolat
PLAT220_ALERT_2_C NonSolvent   Resd 1   C   Ueq(max)/Ueq(min) Range          5.9 Ratio
PLAT222_ALERT_3_C NonSolvent Resd 1   H   Uiso(max)/Uiso(min) Range          9.1 Ratio
PLAT242_ALERT_2_C Low    'MainMol' Ueq as Compared to Neighbors of           Rh1 Check
PLAT242_ALERT_2_C Low    'MainMol' Ueq as Compared to Neighbors of            C1 Check
PLAT242_ALERT_2_C Low    'MainMol' Ueq as Compared to Neighbors of            C2 Check
PLAT242_ALERT_2_C Low    'MainMol' Ueq as Compared to Neighbors of            C3 Check
PLAT242_ALERT_2_C Low    'MainMol' Ueq as Compared to Neighbors of            C4 Check
PLAT242_ALERT_2_C Low    'MainMol' Ueq as Compared to Neighbors of            C5 Check
PLAT905_ALERT_3_C Negative K value in the Analysis of Variance ...       -1.052 Report
PLAT911_ALERT_3_C Missing FCF Refl Between Thmin & STh/L=    0.600           8 Report
PLAT913_ALERT_3_C Missing # of Very Strong Reflections in FCF ....           4 Note
```

Alert level G

```
PLAT154_ALERT_1_G The s.u.'s on the Cell Angles are Equal ..(Note)       0.001 Degree
PLAT187_ALERT_4_G The CIF-Embedded .res File Contains RIGU Records           2 Report
PLAT230_ALERT_2_G Hirshfeld Test Diff for    O11      --C21      .         5.5 s.u.
PLAT232_ALERT_2_G Hirshfeld Test Diff (M-X)  Rh2      --S2       .         5.2 s.u.
PLAT232_ALERT_2_G Hirshfeld Test Diff (M-X)  Co1      --C21      .         7.9 s.u.
PLAT232_ALERT_2_G Hirshfeld Test Diff (M-X)  Co5      --C23      .         6.0 s.u.
PLAT232_ALERT_2_G Hirshfeld Test Diff (M-X)  Co6      --C22      .         6.0 s.u.
PLAT343_ALERT_2_G Unusual sp?      Angle Range in Main Residue for         C28 Check
PLAT606_ALERT_4_G Solvent Accessible VOID(S) in Structure ........           ! Info
PLAT794_ALERT_5_G Tentative Bond Valency for Co1        (III)      .        2.28 Info
PLAT794_ALERT_5_G Tentative Bond Valency for Co2        (III)      .        2.25 Info
PLAT794_ALERT_5_G Tentative Bond Valency for Co3        (III)      .        2.56 Info
PLAT794_ALERT_5_G Tentative Bond Valency for Co5        (III)      .        2.60 Info
PLAT794_ALERT_5_G Tentative Bond Valency for Co6        (III)      .        2.30 Info
PLAT794_ALERT_5_G Tentative Bond Valency for Co7        (III)      .        2.39 Info
PLAT860_ALERT_3_G Number of Least-Squares Restraints ............         150 Note
PLAT869_ALERT_4_G ALERTS Related to the Use of SQUEEZE  Suppressed           ! Info
PLAT883_ALERT_1_G No Info/Value for _atom_sites_solution_primary .      Please Do !
```

```
PLAT912_ALERT_4_G Missing # of FCF Reflections Above STh/L=  0.600          1 Note
PLAT933_ALERT_2_G Number of HKL-OMIT Records in Embedded .res File         23 Note
PLAT967_ALERT_5_G Note: Two-Theta Cutoff Value in Embedded .res ..       56.0 Degree
PLAT978_ALERT_2_G Number C-C Bonds with Positive Residual Density.          3 Info
```

```
   0 ALERT level A = Most likely a serious problem - resolve or explain
   1 ALERT level B = A potentially serious problem, consider carefully
  15 ALERT level C = Check. Ensure it is not caused by an omission or oversight
  22 ALERT level G = General information/check it is not something unexpected

   2 ALERT type 1 CIF construction/syntax error, inconsistent or missing data
  19 ALERT type 2 Indicator that the structure model may be wrong or deficient
   6 ALERT type 3 Indicator that the structure quality may be low
   4 ALERT type 4 Improvement, methodology, query or suggestion
   7 ALERT type 5 Informative message, check
```

It is advisable to attempt to resolve as many as possible of the alerts in all categories. Often the minor alerts point to easily fixed oversights, errors and omissions in your CIF or refinement strategy, so attention to these fine details can be worthwhile. In order to resolve some of the more serious problems it may be necessary to carry out additional measurements or structure refinements. However, the purpose of your study may justify the reported deviations and the more serious of these should normally be commented upon in the discussion or experimental section of a paper or in the "special_details" fields of the CIF. checkCIF was carefully designed to identify outliers and unusual parameters, but every test has its limitations and alerts that are not important in a particular case may appear. Conversely, the absence of alerts does not guarantee there are no aspects of the results needing attention. It is up to the individual to critically assess their own results and, if necessary, seek expert advice.

Publication of your CIF in IUCr journals

A basic structural check has been run on your CIF. These basic checks will be run on all CIFs submitted for publication in IUCr journals (*Acta Crystallographica*, *Journal of Applied Crystallography*, *Journal of Synchrotron Radiation*); however, if you intend to submit to *Acta Crystallographica Section C* or *E* or *IUCrData*, you should make sure that full publication checks are run on the final version of your CIF prior to submission.

Publication of your CIF in other journals

Please refer to the *Notes for Authors* of the relevant journal for any special instructions relating to CIF submission.

PLATON version of 20/01/2022; check.def file version of 19/01/2022

Datablock shelx - ellipsoid plot

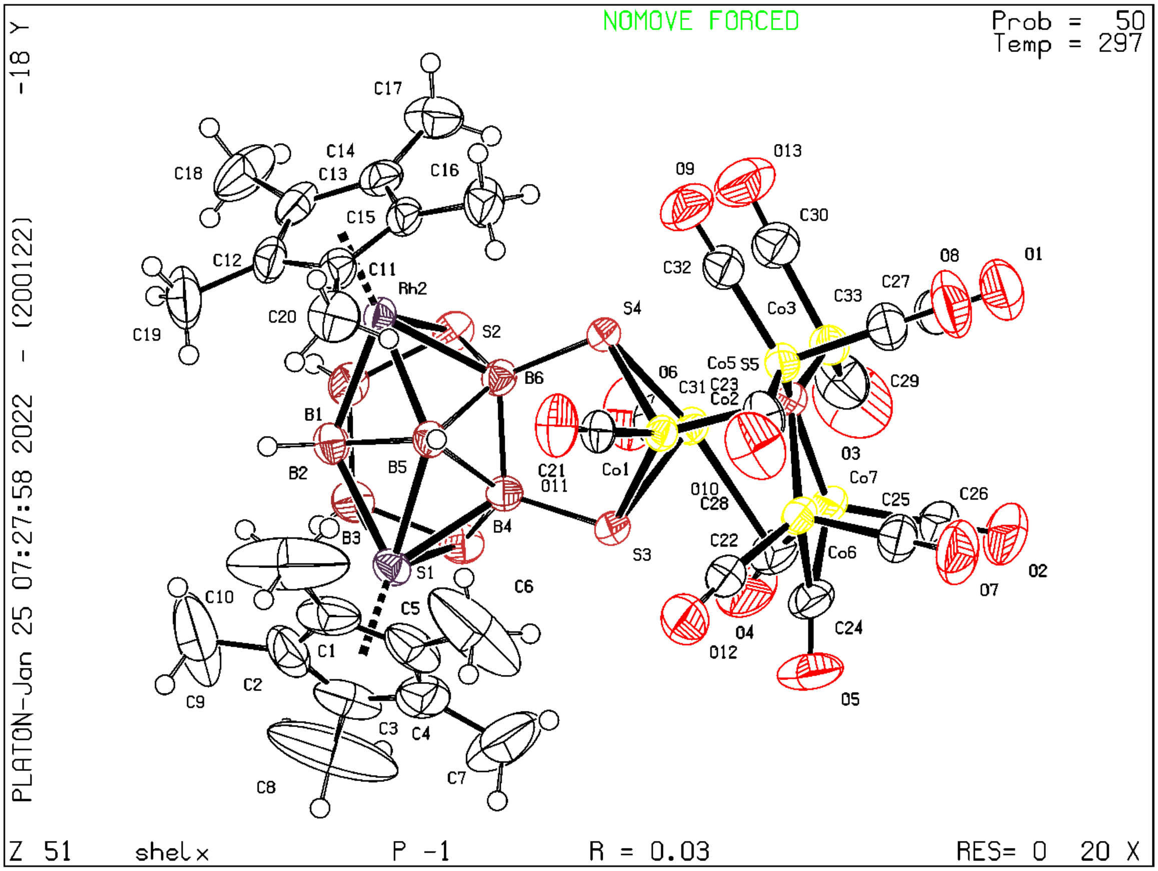

Supplement: Supplementary file 1 [file molecules-28-00998-s001.zip › CheckCIF/checkcif of 8.pdf]
