# Supplementary material for: Chemistry of CS2 and CS3 Bridged Decaborane Analogues: Regular Coordination Versus Cluster Expansion
Source: Molecules. 2023 Jan 19;28(3):998. doi: 10.3390/molecules28030998 (PMC9919328; doi:10.3390/molecules28030998)
Supplement: Supplementary file 1 [file molecules-28-00998-s001.zip › Supporting Information.pdf]

Article

Chemistry of CS_2 and CS_3 Bridged Decaborane Analogues: Regular Coordination Versus Cluster Expansion

Ketaki Kar [1], Suvam Saha [1], Rahul Maganbhai Parmar [1], Arindam Roy [1], Marie Cordier [2], Thierry Roisnel [2] and Sundargopal Ghosh [1,*]

1 Department of Chemistry, Indian Institute of Technology Madras, Chennai 600036, India
2 Univ Rennes, CNRS, Institut des Sciences Chimiques de Rennes, UMR 6226, F-35000 Rennes, France
* Correspondence: sghosh@iitm.ac.in

Table of Contents

I. Supplementary Data

Figure S1 Molecular structure and labelling diagram of **4**

Figure S2 Molecular structure and labelling diagram of **5**

Figure S3 Molecular structure and labelling diagram of **6**

Figure S4 Molecular structure and labelling diagram of **7**

II. Spectroscopic Details

Figure S5 ^{1}H NMR spectrum of compound **3**

Figure S6 $^{11}B\{^1H\}$ NMR spectrum of compound **3**

Figure S7 IR spectrum of **3**

Figure S8 ESI-MS of compound **4**

Figure S9 ^{1}H NMR spectrum of compound **4**

Figure S10 $^{11}B\{^1H\}$ NMR spectrum of compound **4**

Figure S11 IR spectrum of **4**

Figure S12 ESI-MS of compound **5**

Figure S13 ^{1}H NMR spectrum of compound **5**

Figure S14 $^{11}B\{^1H\}$ NMR spectrum of compound **5**

Figure S15 IR spectrum of **5**

Figure S16 ESI-MS of compound **6**

Figure S17 ^{1}H NMR spectrum of compound **6**

Figure S18 $^{11}B\{^1H\}$ NMR spectrum of compound **6**

Figure S19 IR spectrum of **6**

III. Electronic Structure Analysis

Figure S20 HOMO-36 of **7**

Figure S21 HOMO-35 of **8**

Figure S22 Laplacian of the electron density map in the S3-S4-Co1 plane and Co1-Co2-Co3 plane of **8**

Figure S23 Optimized geometry of **4**

Figure S24 Optimized geometry of **5**

Figure S25 Optimized geometry of **6**

Figure S26 Optimized geometry of **7**

Figure S27 Optimized geometry of **8**

I. Supplementary Data

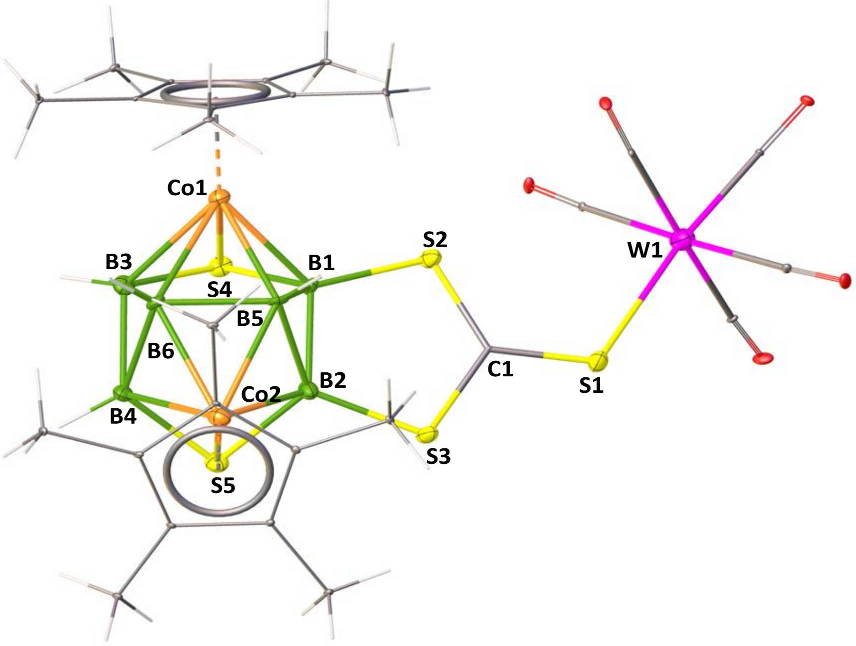

Figure S1. Molecular structures and labelling diagrams of **4**. Selected bond lengths (Å) and angles (°). **4**: B1−B2 1.790(19), W1−S1 2.529(3), S1−C1 1.648(12), S2−B1 1.886(12); C1−S1−W1 116.3(4), C1−S2−B1 102.0(6).

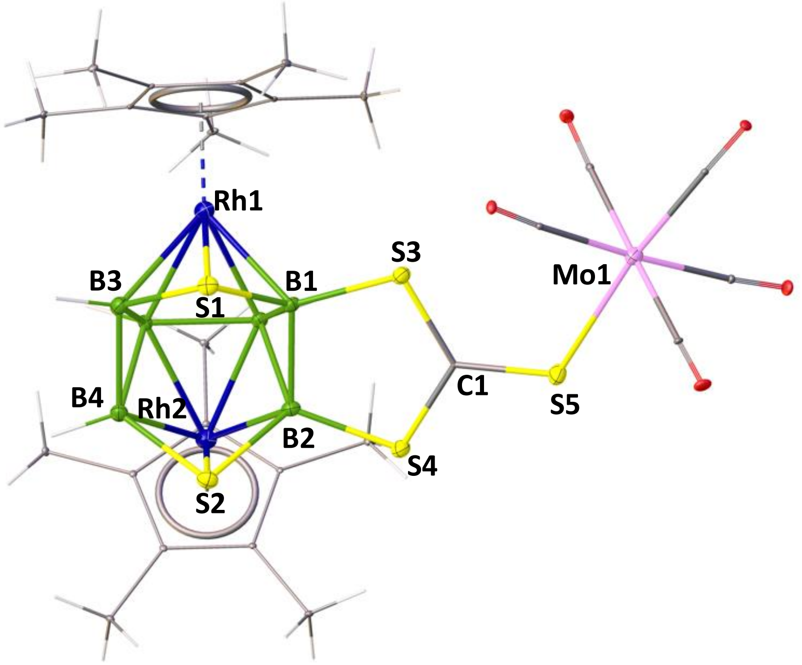

Figure S2. Molecular structures and labelling diagrams of **5**. Selected bond lengths (Å) and angles (°): **5**: B1−B2 1.808(8), Mo1−S5 2.5388(15), S5−C1 1.668(6), S3−B1 1.869(6); C1−S5−Mo1 117.1(2), C1−S3−B1 101.1(3).

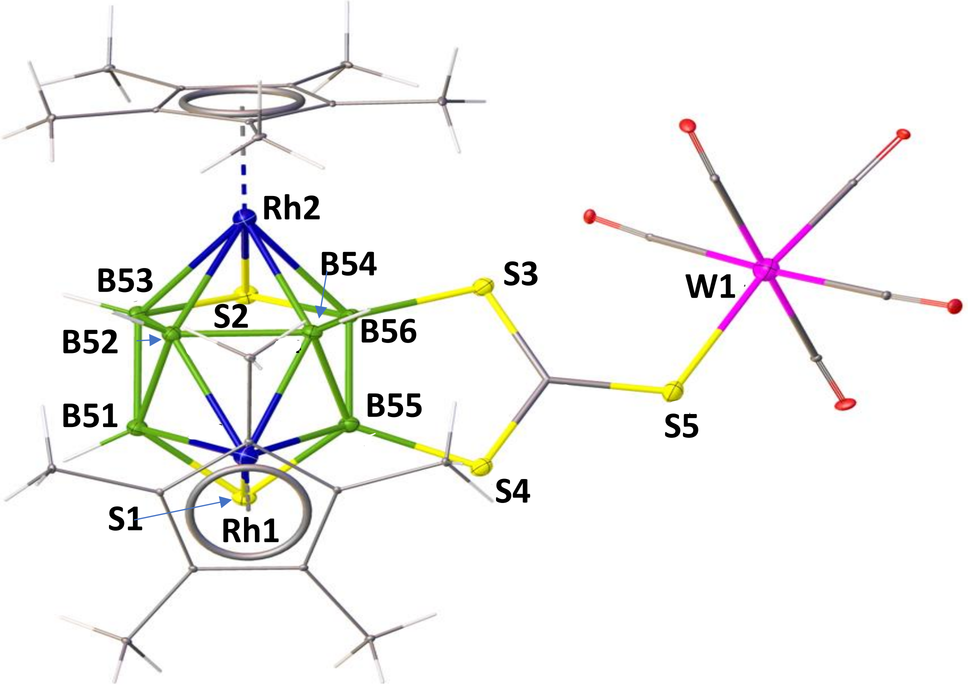

Figure S3. Molecular structures and labelling diagrams of **6**. Selected bond lengths (Å) and angles (°): **6**: B5−B6 1.809(4), W1−S5 2.5293(7), S5−C1 1.672(3), S3−B6 1.873(3); C1−S5−W1 116.73(9), C1−S3−B6 101.03(12).

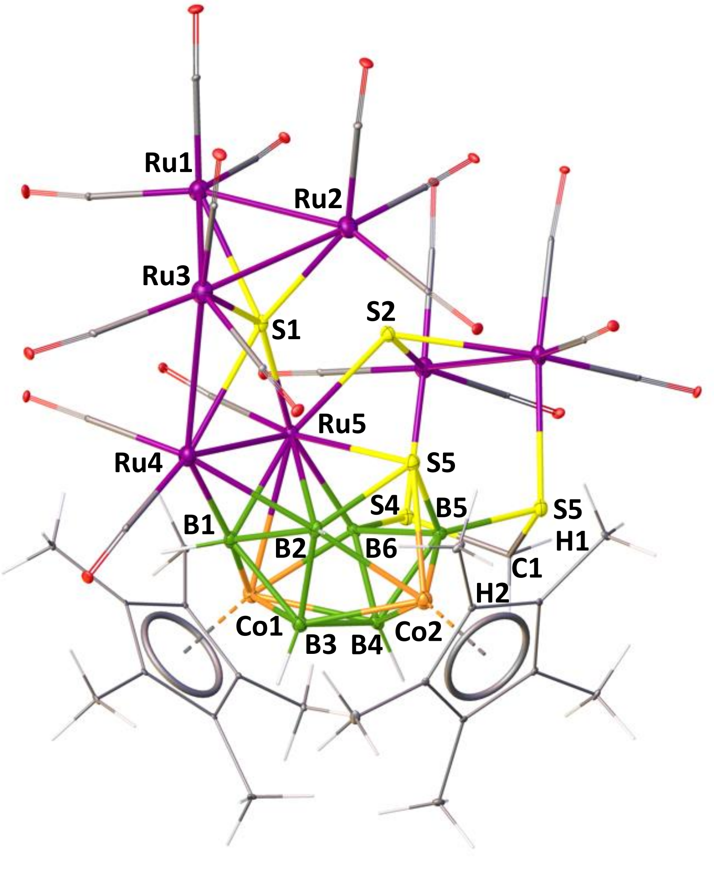

Figure S4. Molecular structure and labelling diagram of **7**. Selected bond lengths (Å) and angles (deg) in **7**: Ru1–S1 2.3488(18), Ru3–S1 2.4798(18), Ru4–S1 2.5153(18), Ru3–Ru4 3.0572(8),Ru7–S2 2.4311(17), S5–C7 1.821(7); Ru5–S1–Ru3 145.51(8), Ru4–S1–Ru3 75.47(5).

II. Spectroscopic details

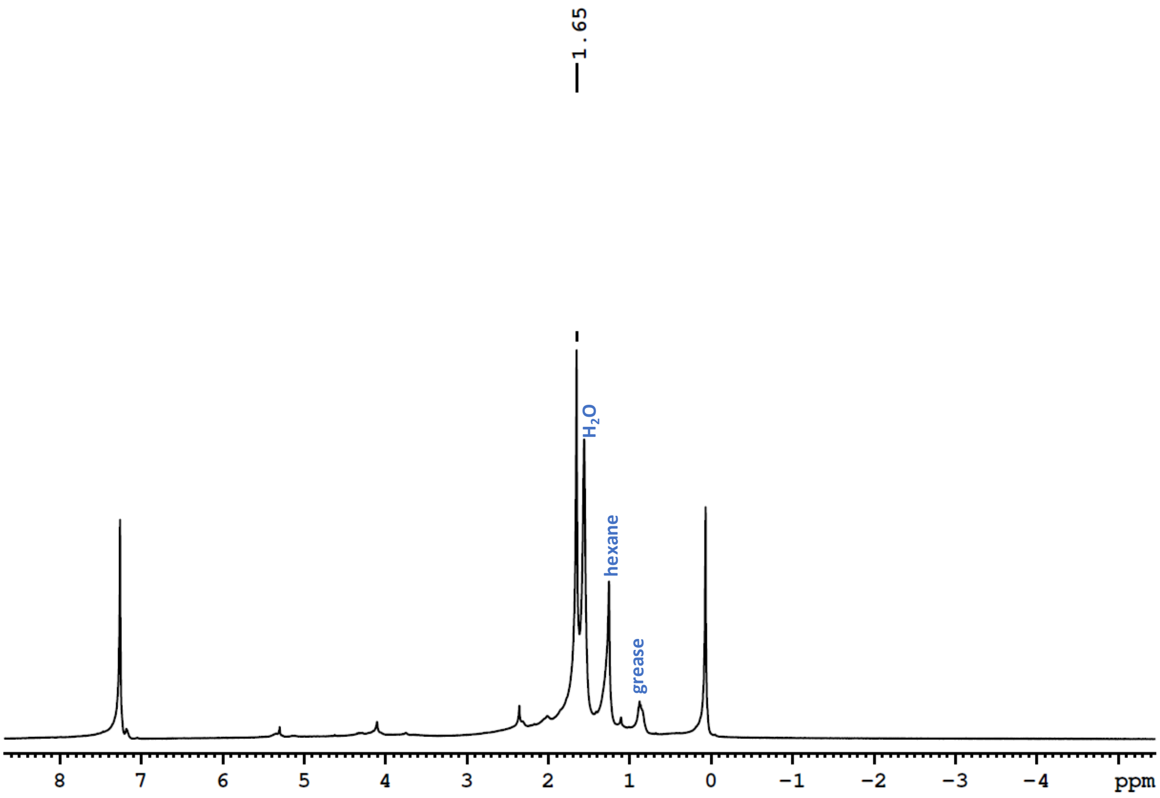

Figure S5. ^{1}H NMR spectrum of compound **3** in $CDCl_3$.

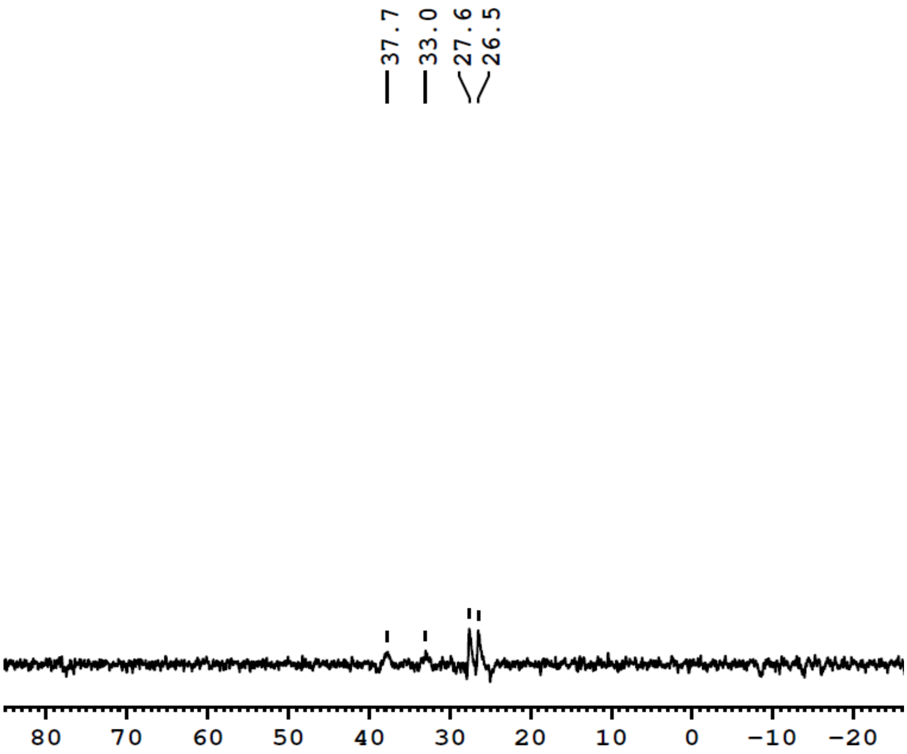

Figure S6. $^{11}B\{^{1}H\}$ NMR spectrum of compound **3** in C_6D_6. [1-3]

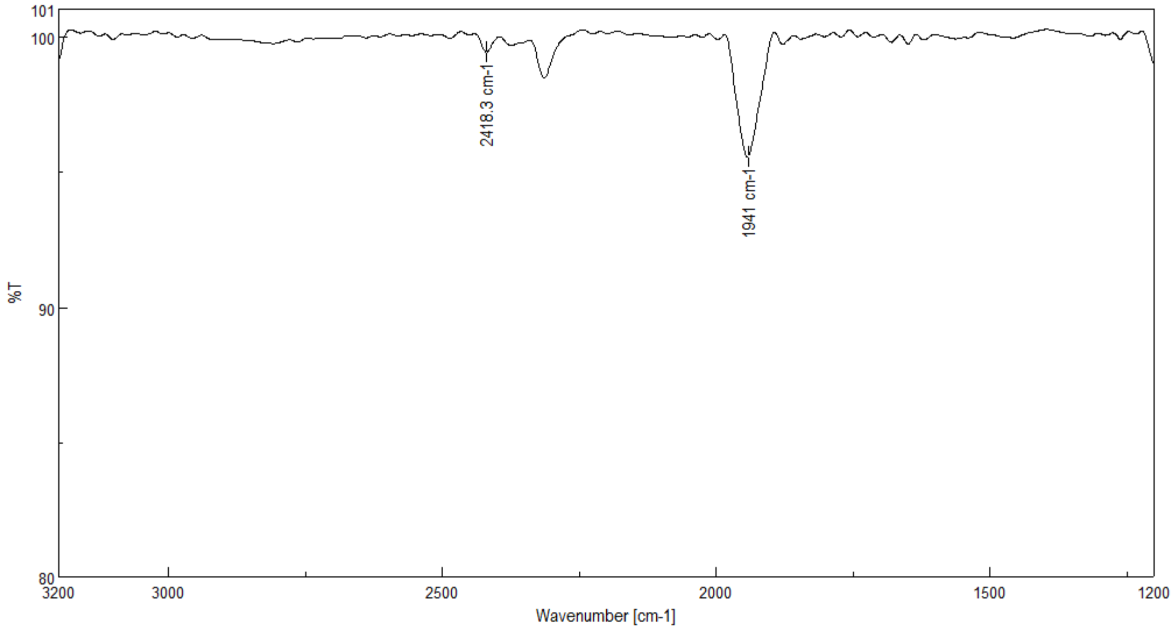

Figure S7. IR spectrum of **3** in CH_2Cl_2.

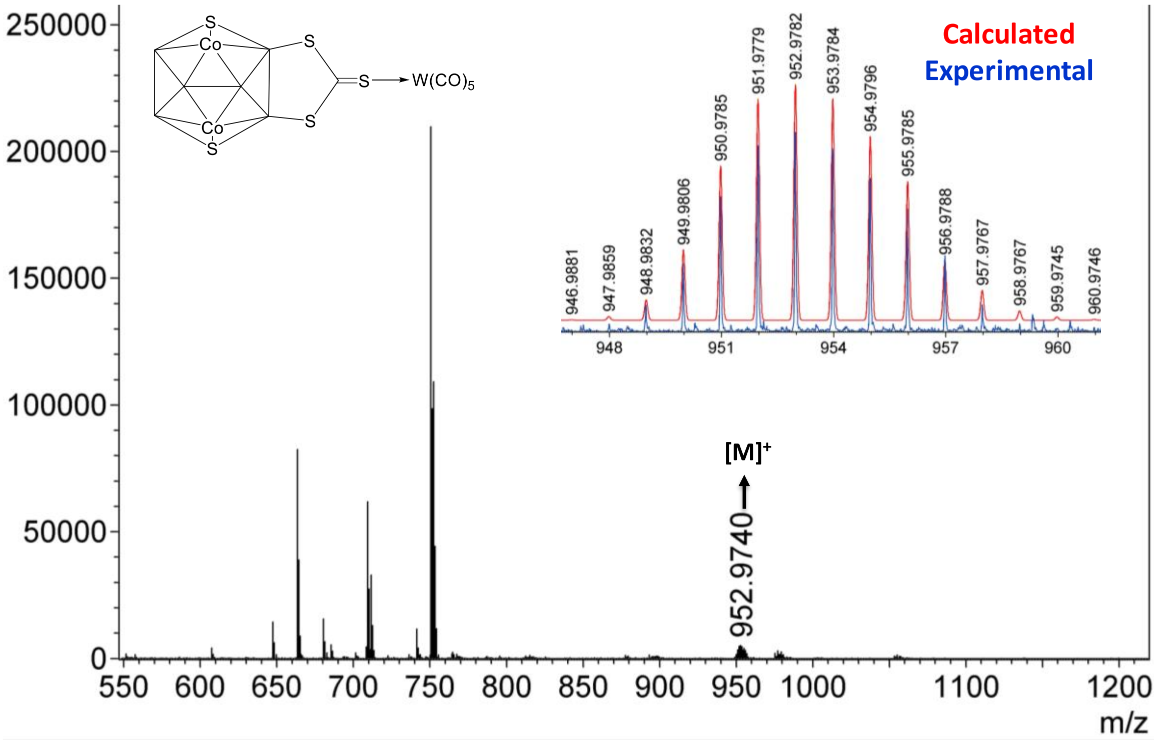

Figure S8. ESI-MS of compound **4**.

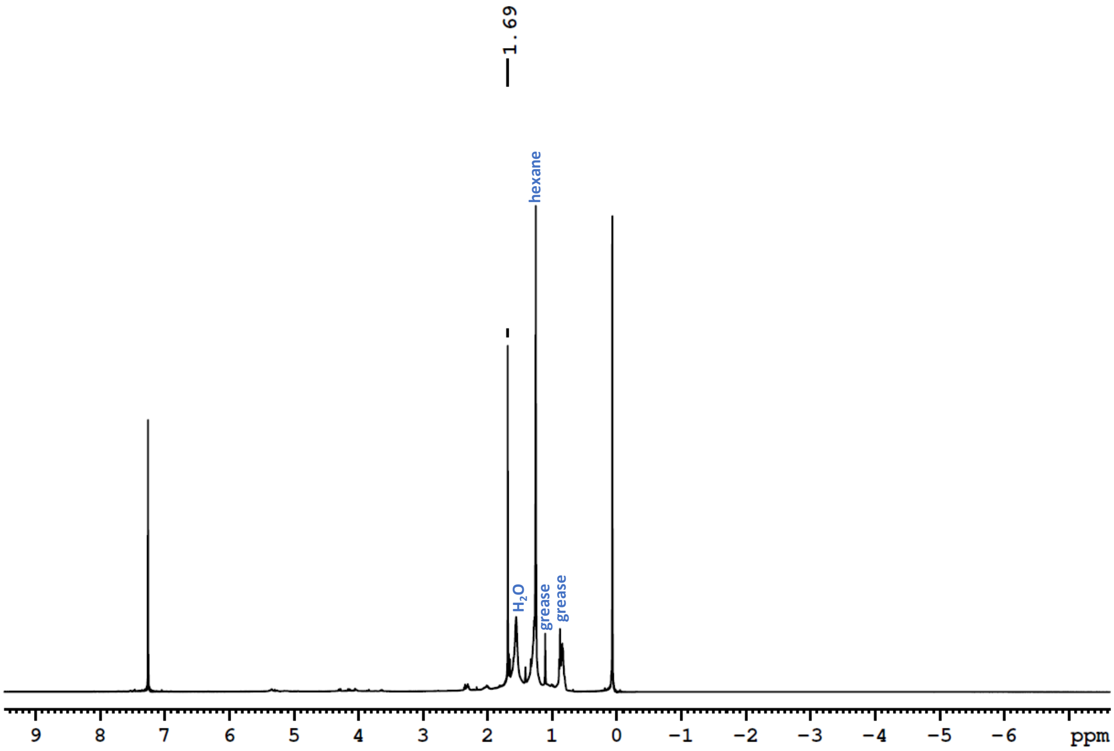

Figure S9. ^{1}H NMR spectrum of compound **4** in $CDCl_3$.

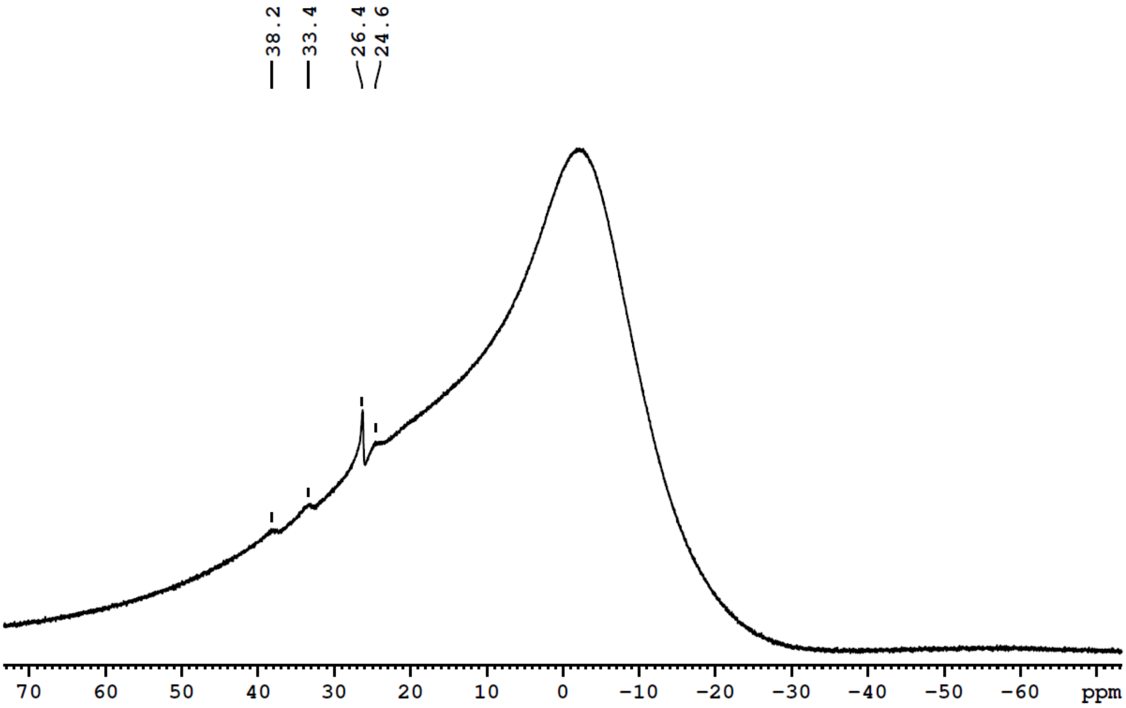

Figure S10. $^{11}B\{^{1}H\}$ NMR spectrum of compound **4** in $CDCl_3$.

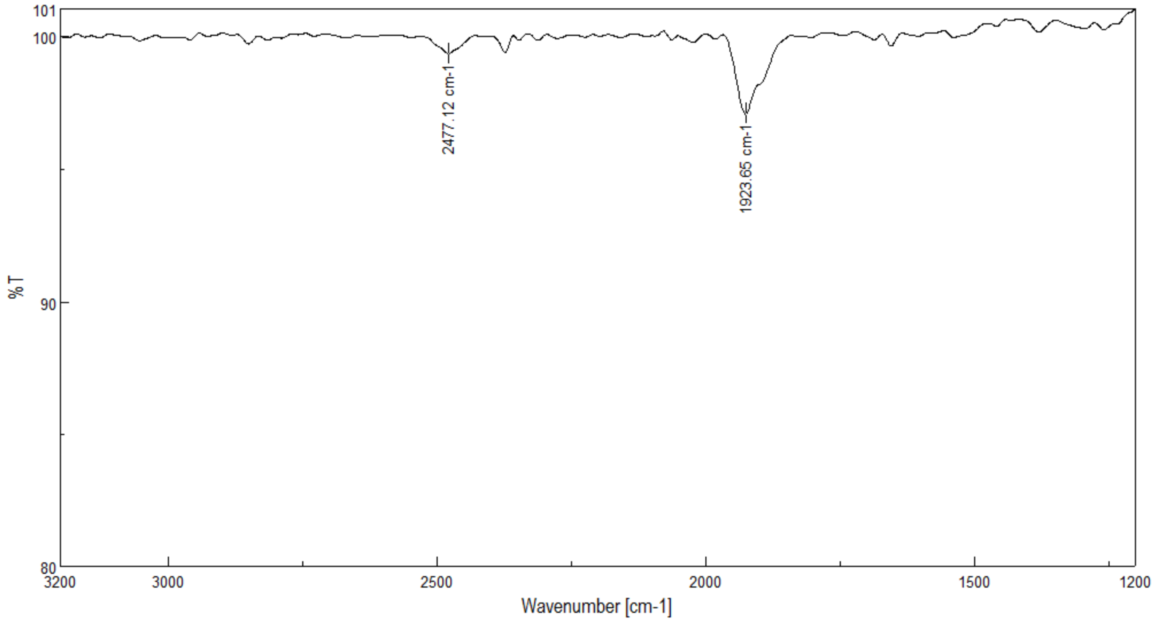

Figure S11. IR spectrum of **4** in CH_2Cl_2.

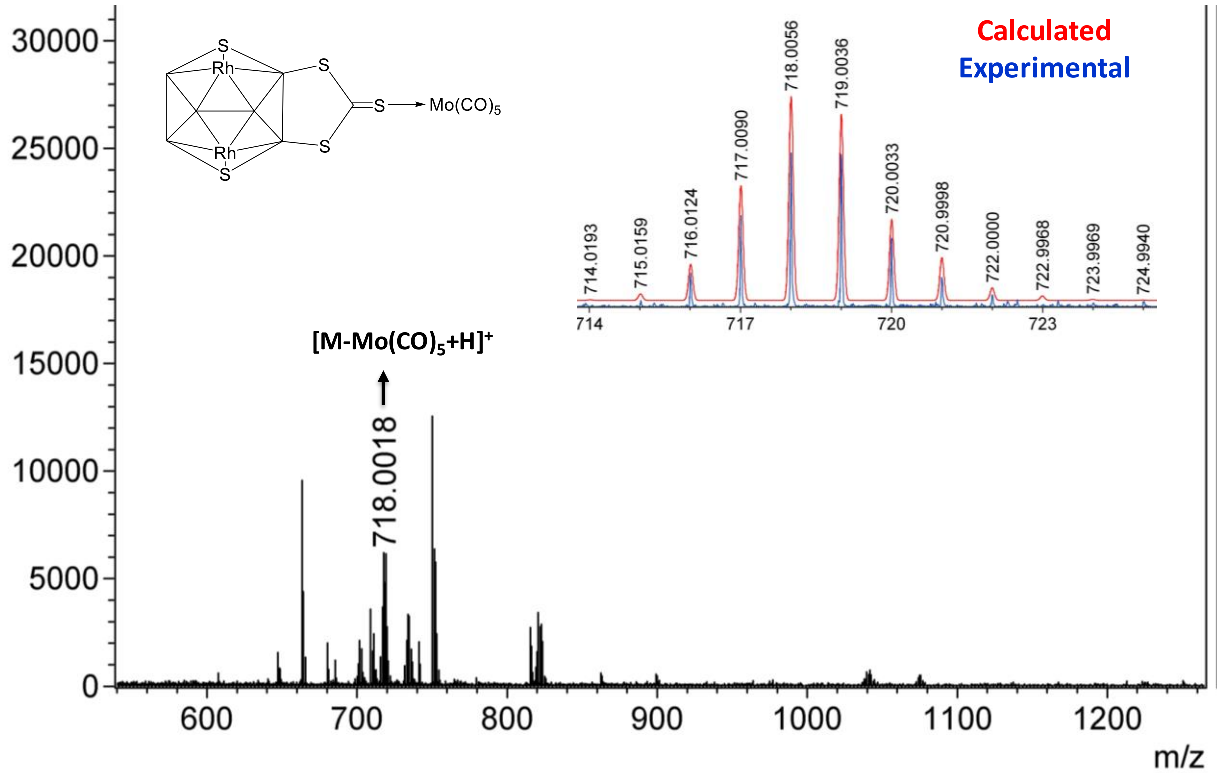

Figure S12. ESI-MS of compound **5.**

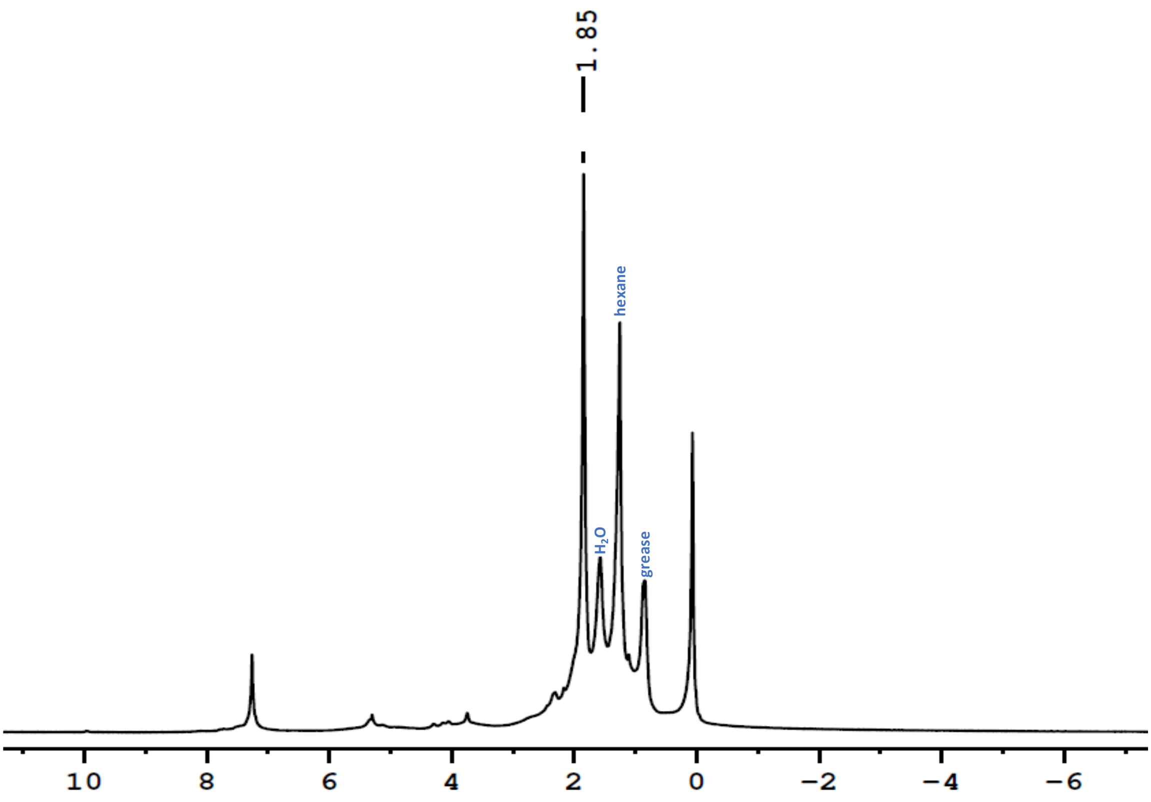

Figure S13. ^{1}H NMR spectrum of compound **5** in $CDCl_3$.

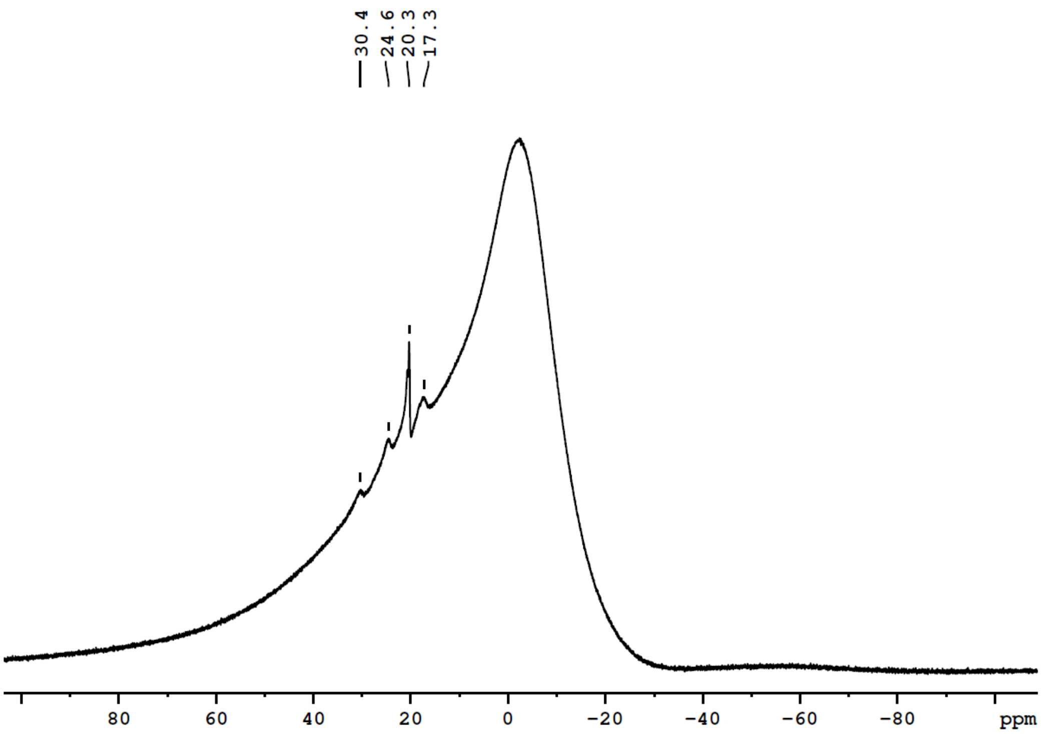

Figure S14. $^{11}B\{^{1}H\}$ NMR spectrum of compound **5** in $CDCl_3$.

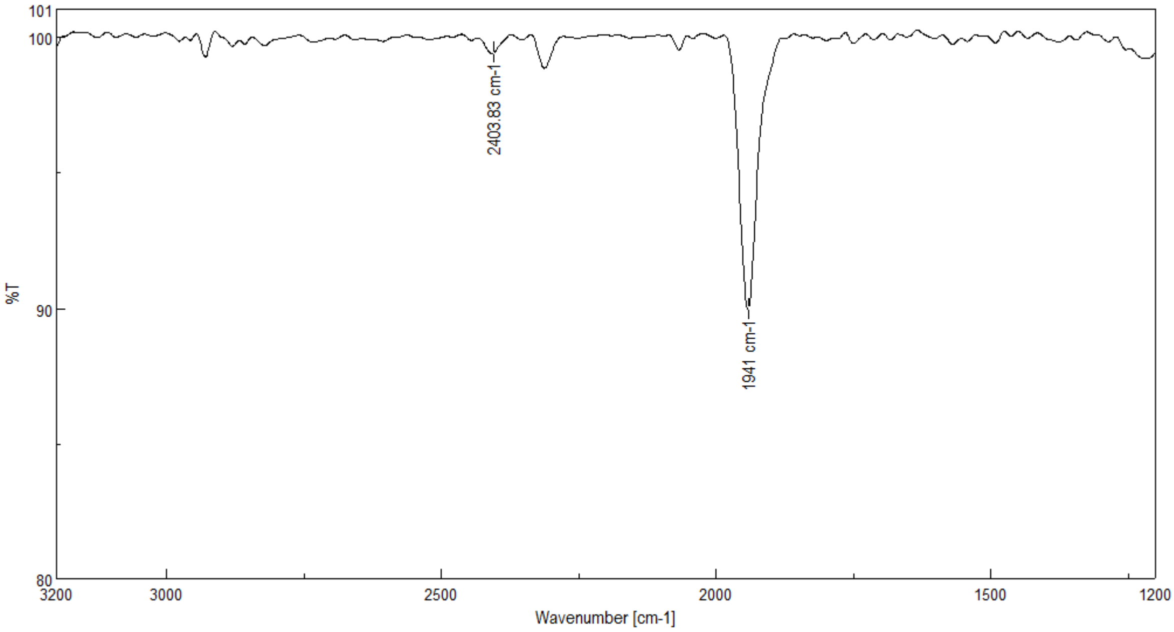

Figure S15. IR spectrum of **5** in CH_2Cl_2.

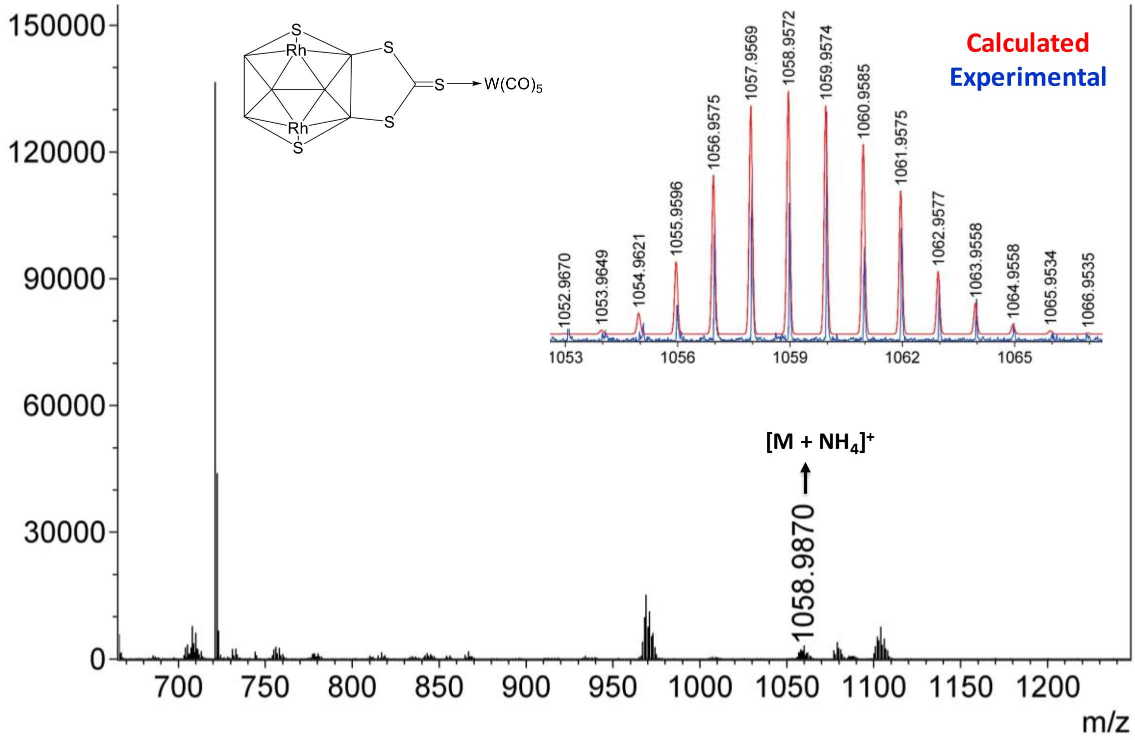

Figure S16. ESI-MS of compound **6.**

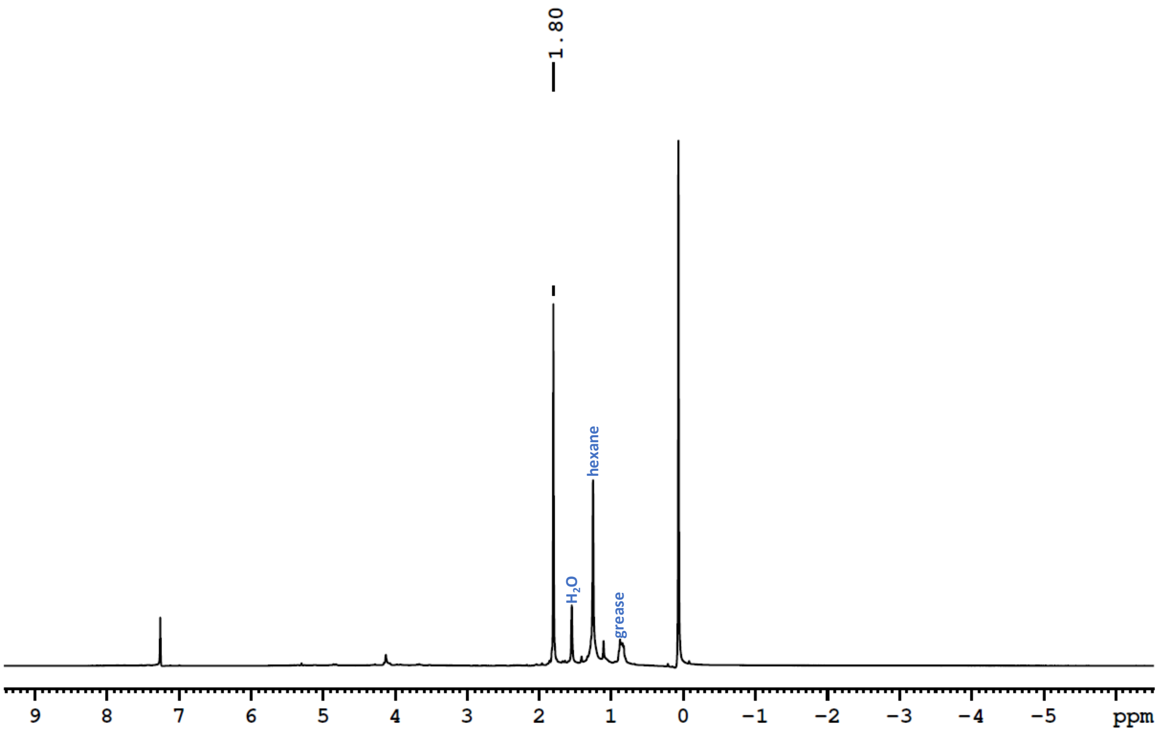

Figure S17. ^1H NMR spectrum of compound **6** in $CDCl_3$.

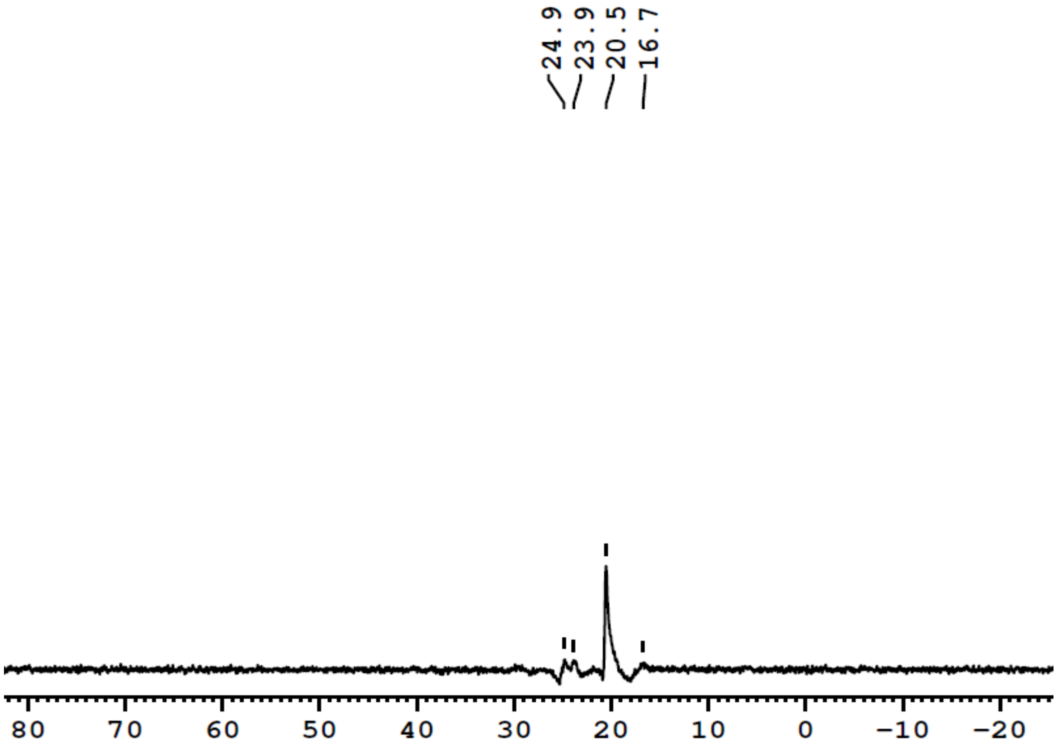

Figure S18. $^{11}B\{^1H\}$ NMR spectrum of compound **6** in C_6D_6.[1-3]

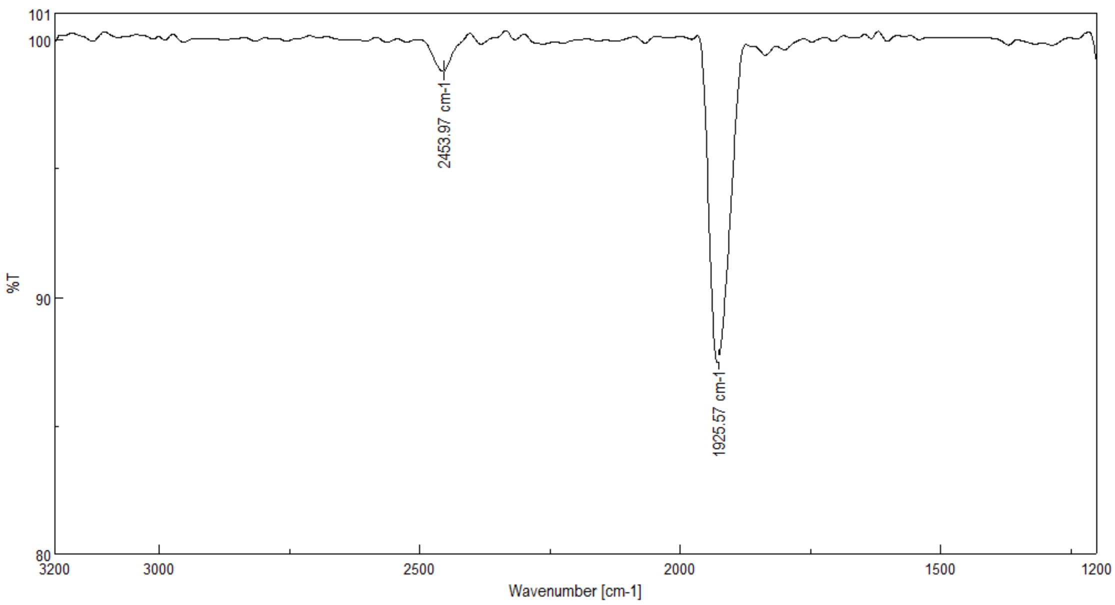

Figure S19. IR spectrum of **6** in CH_2Cl_2.

III. Electronic Structure Analysis

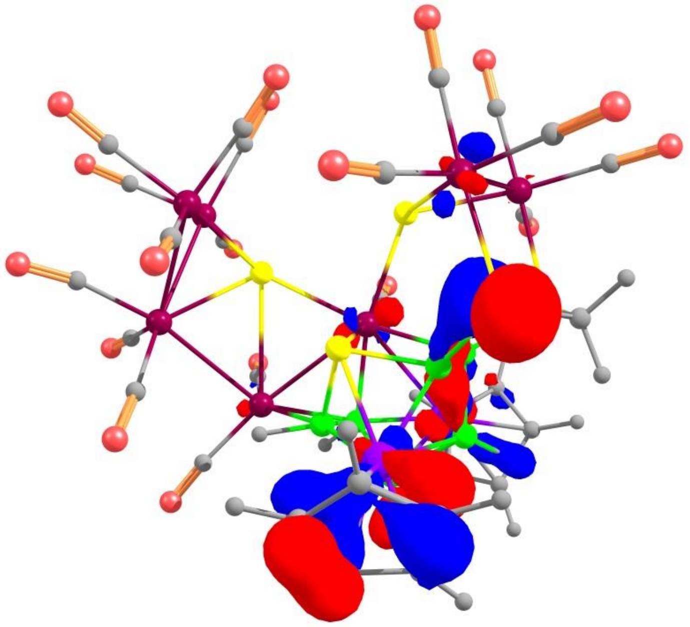

Figure S20. HOMO-36 of **7**. (contour values for isosurface = ± 0.04 [e/bohr^3]$^{1/2}$).

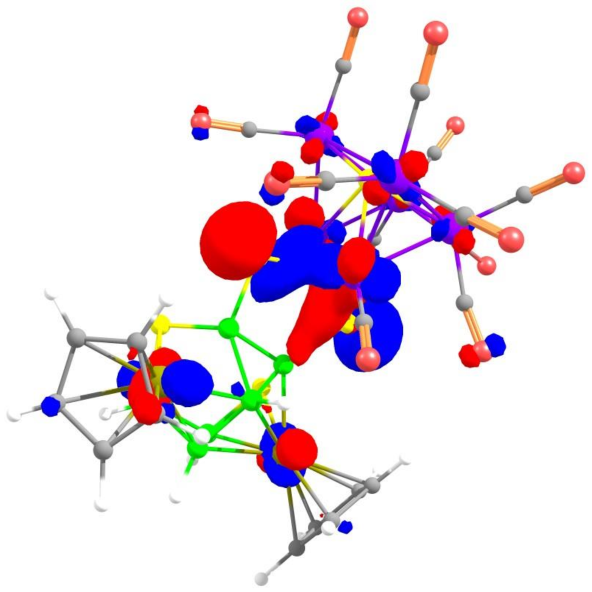

Figure S21. HOMO-35 of **8**. (contour values for isosurface = ± 0.04 [e/bohr^3]$^{1/2}$).

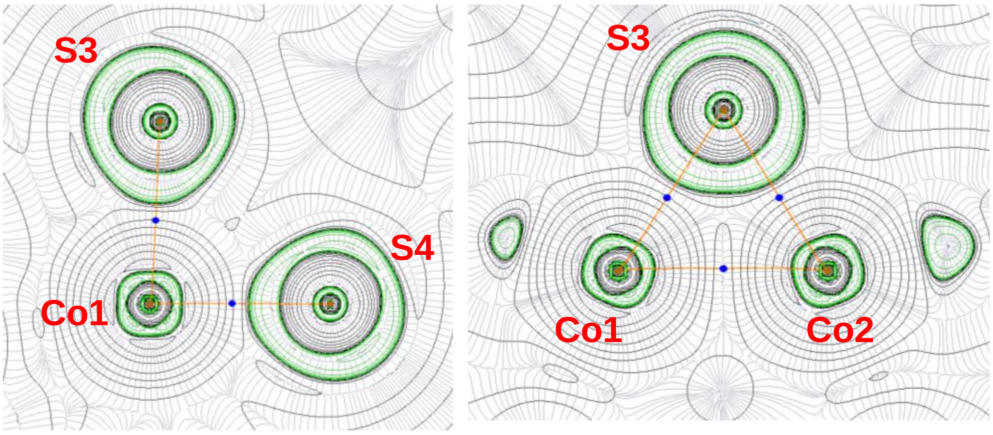

Figure S22. Contour-line map of the Laplacian of the electron density in the S3-S4-Co1 plane (left) and Co1-Co2-S3 plane (right) of **8**. The solid orange lines are bond paths, whereas the blue spheres indicate the ring-critical points. Areas of charge concentration [ρ(r)<0] are meant for green lines and areas of charge depletion [$\rho(r)$>0] are shown by black lines.

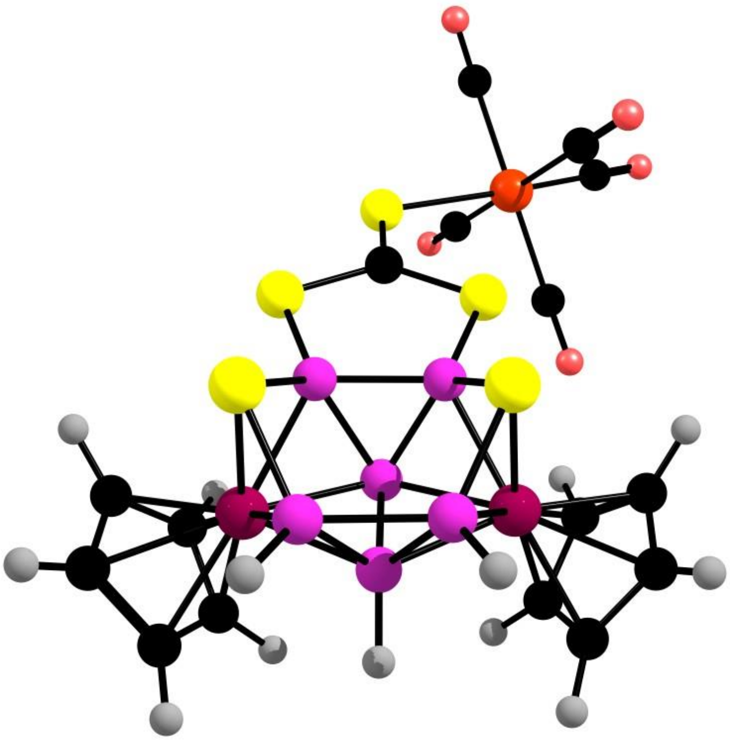

Figure S23. Optimized geometry of **4**

Total energy = -5966.4520685 a.u.

Cartesian coordinates for the calculated structure **4** (in Å)

W	4.003176000	-0.155524000	0.099499000
Co	-2.661326000	2.088568000	0.076945000
Co	-4.030690000	-1.401477000	0.245654000
S	2.098042000	-1.873149000	-0.272324000
S	0.315349000	0.504447000	-0.788481000
S	-0.790140000	-2.332125000	-0.677990000
S	-2.376220000	1.709757000	-2.103450000
S	-3.639000000	-1.500162000	-1.949879000
O	4.846123000	-1.892150000	2.695291000
O	5.894091000	-2.047203000	-1.724961000
O	3.402784000	1.680593000	-2.499696000
O	2.219602000	1.755568000	2.002397000
O	6.507868000	1.717585000	0.681035000
C	4.523403000	-1.281338000	1.759987000
C	5.200462000	-1.377044000	-1.076622000
C	3.574878000	1.005255000	-1.568908000
C	2.837491000	1.059577000	1.299266000
C	5.588303000	1.029435000	0.465731000
C	-1.349263000	2.899259000	1.514184000
C	-2.690786000	2.774015000	2.021285000
C	-3.561741000	3.595782000	1.213460000
C	-2.766750000	4.192834000	0.191385000
C	-1.400902000	3.758627000	0.375391000
C	-3.595674000	-2.648813000	1.875180000
C	-4.124194000	-3.434829000	0.807727000
C	-5.449328000	-2.946985000	0.500574000
C	-5.739149000	-1.859718000	1.376434000
C	-4.582576000	-1.655086000	2.214373000
C	0.574402000	-1.215772000	-0.567452000
B	-1.552132000	0.568625000	-0.850411000
B	-2.213210000	-1.125839000	-0.773676000
B	-4.107806000	1.580841000	-1.372640000
H	-4.896215000	2.397466000	-1.800047000
B	-4.796683000	-0.170071000	-1.286836000
H	-5.944580000	-0.267073000	-1.666542000
B	-2.569070000	0.055772000	0.539461000
H	-1.948254000	-0.137743000	1.566467000
B	-4.228160000	0.690248000	0.162957000
H	-5.136312000	1.077179000	0.877373000
H	-2.595785000	-2.745053000	2.316178000
H	-3.602416000	-4.247908000	0.286493000
H	-4.476075000	-0.880220000	2.983492000
H	-6.653158000	-1.253591000	1.378320000
H	-6.100349000	-3.319938000	-0.300558000
H	-4.646609000	3.700368000	1.335476000
H	-2.997690000	2.166305000	2.881410000
H	-0.454691000	2.390087000	1.895500000
H	-0.552628000	4.019658000	-0.270813000
H	-3.134225000	4.833214000	-0.620624000

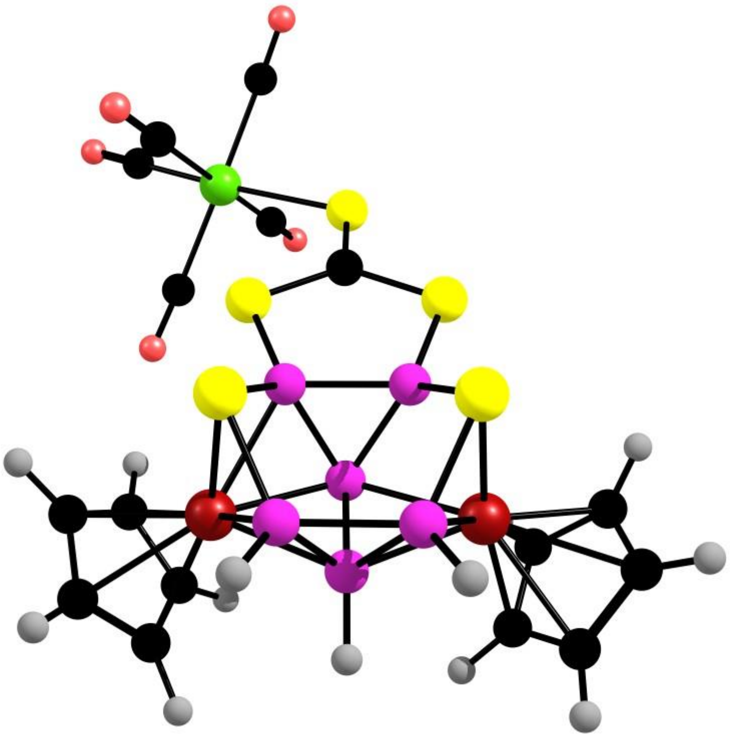

Figure S24. Optimized geometry of **5**

Total energy = -3423.2055592 a.u.

Cartesian coordinates for the calculated structure **2** (in Å)

Rh	-1.956446000	2.184854000	-0.017153000	C	3.518207000	1.038762000	-1.207034000
Rh	-3.489231000	-1.463074000	-0.181942000	C	6.247220000	0.841130000	-0.476762000
Mo	4.648038000	-0.304116000	-0.147689000	C	5.054370000	-1.317460000	-1.888534000
S	-1.611289000	1.654243000	2.253258000	C	5.838020000	-1.626772000	0.887678000
S	-2.940696000	-1.516594000	2.110771000	B	-0.860770000	0.518866000	0.933029000
S	1.002607000	0.414453000	0.812777000	B	-1.560681000	-1.164518000	0.856575000
S	-0.164707000	-2.396579000	0.686005000	B	-3.378855000	1.583654000	1.586022000
S	2.720480000	-1.983037000	0.199949000	H	-4.134891000	2.396639000	2.072482000
O	4.183275000	1.341259000	2.581516000	B	-4.105032000	-0.152145000	1.508214000
O	2.933701000	1.827528000	-1.836042000	H	-5.231280000	-0.219927000	1.951469000
O	7.183402000	1.511660000	-0.670278000	B	-1.936896000	0.045382000	-0.452479000
O	5.312637000	-1.869692000	-2.877807000	H	-1.333501000	-0.160228000	-1.487169000
O	6.539988000	-2.352624000	1.461381000	B	-3.608833000	0.728075000	0.023426000
C	-0.505128000	3.259972000	-1.437692000	H	-4.565279000	1.148110000	-0.603415000
C	-0.851606000	4.165596000	-0.384054000	H	-2.360877000	-2.543055000	-2.723764000
C	-2.274130000	4.414436000	-0.431403000	H	-2.949964000	-4.387246000	-0.809940000
C	-2.816051000	3.631660000	-1.496585000	H	-5.409543000	-3.784183000	0.214688000
C	-1.723843000	2.897120000	-2.108183000	H	-6.366415000	-1.596904000	-1.090903000
C	-3.281794000	-2.588397000	-2.129051000	H	-4.465029000	-0.803391000	-2.885597000
C	-4.387353000	-1.656576000	-2.199801000	H	0.499848000	2.880258000	-1.665086000
C	-5.401603000	-2.087678000	-1.269020000	H	-0.150459000	4.602340000	0.339537000
C	-4.896862000	-3.236137000	-0.586707000	H	-2.838034000	5.072751000	0.241274000
C	-3.590782000	-3.553204000	-1.123282000	H	-3.869798000	3.588193000	-1.798959000
C	1.220959000	-1.306557000	0.555686000	H	-1.807273000	2.219716000	-2.967304000
C	4.308604000	0.733820000	1.599188000				

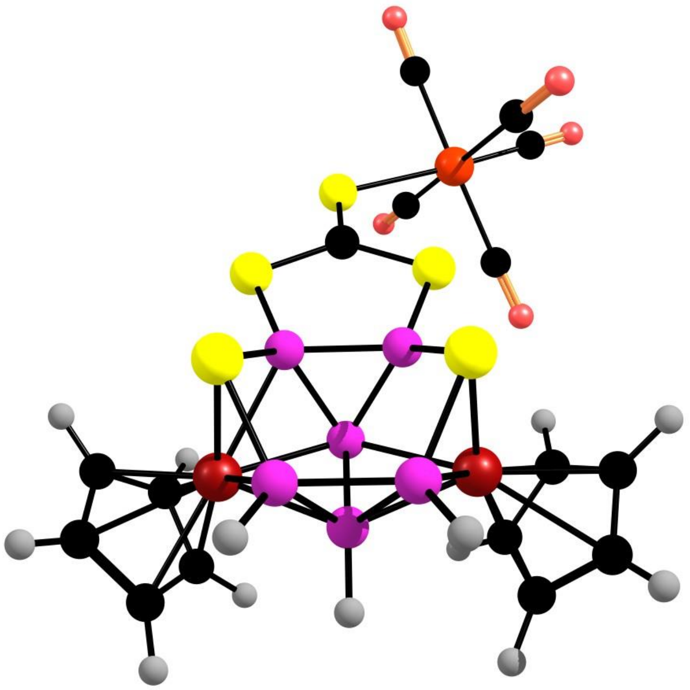

Figure S25. Optimized geometry of **6**.

Total energy = -3422.1091383 a.u.

Cartesian coordinates for the calculated structure **2** (in Å)

Rh	3.832938000	-1.472365000	-0.184286000	B	3.964712000	0.718129000	0.018083000
C	5.658437000	-1.947748000	-1.450432000	H	4.920117000	1.132688000	-0.613859000
C	4.493764000	-1.744302000	-2.283722000	B	3.746242000	1.576336000	1.580091000
C	3.551112000	-2.803302000	-2.009824000	H	4.509347000	2.384756000	2.062908000
C	4.101690000	-3.612358000	-0.970562000	B	2.287692000	0.044363000	-0.451068000
C	5.406344000	-3.085009000	-0.627285000	H	1.681617000	-0.163023000	-1.484007000
Rh	2.319608000	2.183961000	-0.018052000	B	1.908323000	-1.160827000	0.860833000
C	2.658740000	4.408252000	-0.441403000	B	1.219578000	0.525966000	0.938680000
C	1.234213000	4.176940000	-0.374516000	C	-0.872642000	-1.285840000	0.571289000
C	0.862129000	3.273414000	-1.421581000	S	3.289838000	-1.521755000	2.110385000
C	2.067043000	2.894417000	-2.107594000	S	1.982048000	1.658381000	2.254552000
C	3.176596000	3.616106000	-1.511894000	S	-0.645417000	0.432793000	0.828336000
W	-4.300134000	-0.238970000	-0.118699000	S	0.504373000	-2.385584000	0.695554000
C	-3.146630000	1.104944000	-1.184132000	S	-2.379827000	-1.952621000	0.218844000
O	-2.548337000	1.887755000	-1.809739000	H	0.548268000	4.623510000	0.357641000
C	-4.731233000	-1.249175000	-1.875993000	H	-0.150551000	2.906050000	-1.635231000
O	-4.999809000	-1.797330000	-2.865747000	H	3.239795000	5.061035000	0.222017000
C	-5.901840000	0.934993000	-0.441552000	H	4.225550000	3.559036000	-1.828401000
O	-6.832617000	1.616176000	-0.629931000	H	2.130397000	2.214236000	-2.966228000
C	-5.513142000	-1.564193000	0.921622000	H	6.556758000	-1.318194000	-1.432704000
O	-6.220597000	-2.287520000	1.493401000	H	4.373841000	-0.956438000	-3.038225000
C	-3.936007000	0.794507000	1.646211000	H	2.570854000	-2.934539000	-2.485073000
O	-3.792512000	1.393776000	2.632088000	H	3.621045000	-4.485866000	-0.511179000
B	4.460288000	-0.165330000	1.502253000	H	6.079266000	-3.486490000	0.141417000
H	5.588115000	-0.240150000	1.940364000				

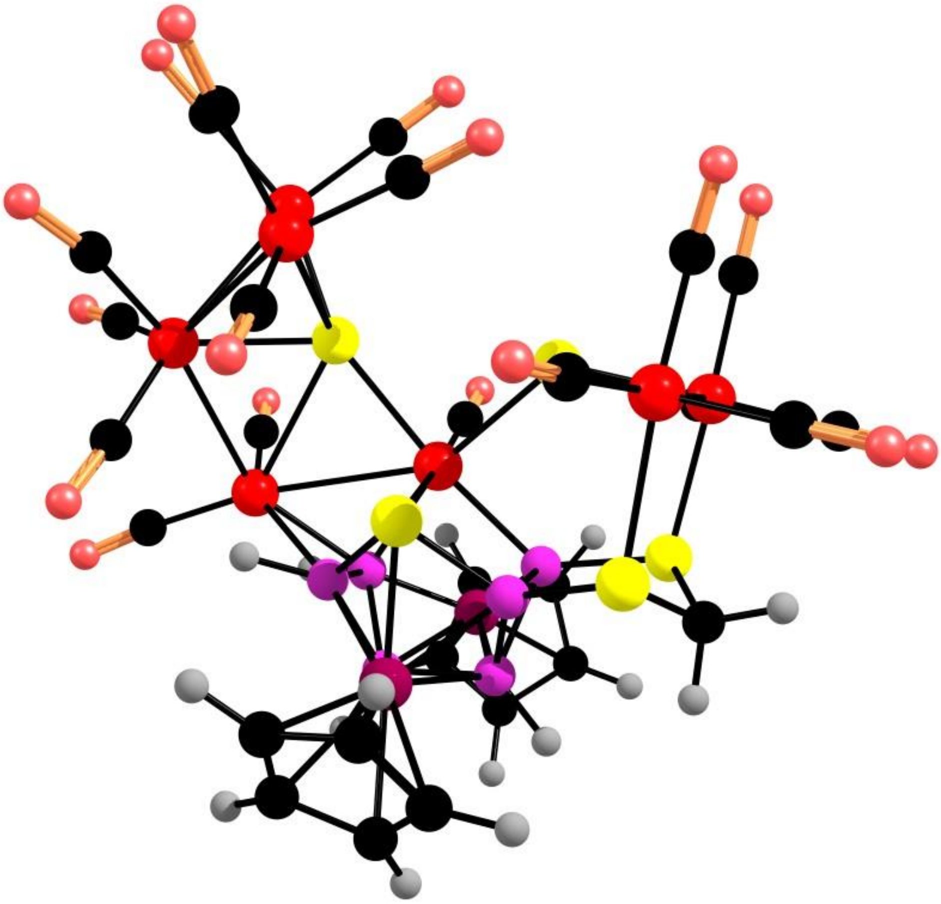

Figure S26. Optimized geometry of **7.**

Total energy = -8038.5743591 a.u.

Cartesian coordinates for the calculated structure **7** (in Å)

Ru	3.310310000	-1.814591000	-1.361716000
C	3.660316000	-1.179783000	-3.131906000
O	3.883984000	-0.930488000	-4.245702000
C	4.974558000	-2.695846000	-1.173584000
O	5.999877000	-3.238413000	-1.083484000
C	2.362070000	-3.362342000	-1.883058000
O	1.788762000	-4.317688000	-2.218478000
Ru	2.443843000	-2.023837000	1.301468000
C	3.765134000	-3.309904000	1.747058000
O	4.517816000	-4.145138000	2.043027000
C	1.156670000	-3.377646000	1.203042000
O	0.381780000	-4.247739000	1.198926000
C	2.007093000	-1.395060000	3.058390000
O	1.807833000	-1.047503000	4.151397000
Ru	3.781739000	0.365005000	0.453114000
C	5.167701000	0.822016000	-0.778229000
O	6.091424000	1.117494000	-1.425718000
C	4.996418000	-0.698368000	1.453952000
O	5.878219000	-1.143048000	2.075775000
C	3.689091000	1.564832000	1.926289000
O	3.714900000	2.283023000	2.851092000
Ru	2.103855000	2.460120000	-0.640939000
C	3.489216000	3.662851000	-0.311500000
O	4.286789000	4.488025000	-0.118262000
C	2.791980000	2.114637000	-2.326651000
O	3.180261000	1.961078000	-3.411014000
Ru	-0.433574000	0.824080000	-0.776067000
C	-0.331122000	0.499709000	-2.595451000
O	-0.247363000	0.265788000	-3.734872000
Ru	-3.782441000	-1.719845000	-1.400373000
C	-3.395200000	-3.506292000	-1.895011000
O	-3.176897000	-4.578051000	-2.276232000
C	-3.704642000	-1.179553000	-3.232434000
O	-3.707165000	-0.866734000	-4.351296000
C	-5.650916000	-2.085600000	-1.223257000
O	-6.788152000	-2.303890000	-1.120469000
Ru	-3.007203000	-2.086635000	1.325267000
C	-2.674025000	-3.893420000	0.919226000

O	-2.520033000	-5.028399000	0.748525000
C	-4.702499000	-2.572124000	2.039059000
O	-5.735013000	-2.867977000	2.484911000
C	-1.938947000	-2.174905000	2.918994000
O	-1.362673000	-2.322188000	3.915500000
Co	-1.846506000	3.056408000	-1.436771000
C	-1.524357000	4.694644000	-2.742633000
C	-2.811553000	4.792444000	-2.111500000
C	-3.584880000	3.644584000	-2.494423000
C	-2.766244000	2.820612000	-3.332570000
C	-1.491035000	3.470775000	-3.486093000
Co	-1.282522000	2.979778000	2.267712000
C	-0.234345000	3.459916000	4.035947000
C	-1.435196000	2.722492000	4.357921000
C	-2.558110000	3.455956000	3.867227000
C	-2.052997000	4.638964000	3.215248000
C	-0.616823000	4.652953000	3.351193000
S	1.665499000	-0.470665000	-0.328406000
S	-1.531253000	-1.461271000	-0.545823000
S	-0.499562000	0.941442000	1.713737000
S	-4.203550000	0.666912000	-0.879654000
S	-3.636571000	0.275755000	2.051180000
B	-0.004358000	2.938885000	-0.817503000
H	0.970011000	3.605599000	-1.415715000
B	0.243462000	2.586681000	0.861586000
H	1.469595000	2.671779000	1.192552000
B	-0.972634000	3.802370000	0.368186000
H	-0.766687000	5.002633000	0.403486000
B	-2.562426000	2.977788000	0.557386000
H	-3.650067000	3.503875000	0.734035000
B	-2.300332000	1.423667000	1.352664000
B	-2.510265000	1.489288000	-0.458829000
C	-4.913248000	0.745723000	0.813299000
H	-5.770450000	0.052040000	0.892715000
H	-5.239658000	1.785414000	1.014976000
H	-4.608577000	3.418766000	-2.172113000
H	-3.140894000	5.598940000	-1.444401000
H	-0.704162000	5.416629000	-2.645638000
H	-3.056277000	1.865808000	-3.788312000
H	-0.642787000	3.091085000	-4.068854000
H	0.793796000	3.141298000	4.253702000
H	-1.469278000	1.750433000	4.867654000
H	-3.611206000	3.159090000	3.944681000
H	-2.657242000	5.403563000	2.711376000
H	0.060649000	5.420023000	2.957017000

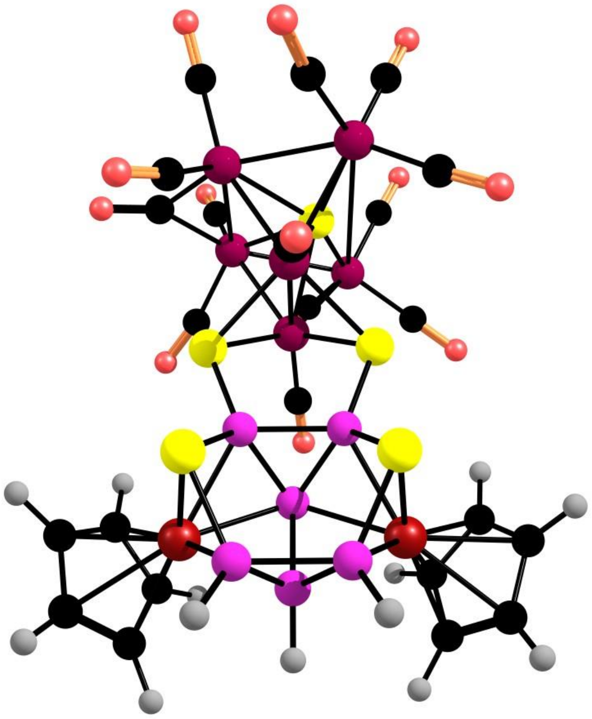

Figure S27. Optimized geometry of **8.**

Total energy = -12520.3674633 a.u.

Cartesian coordinates for the calculated structure **8** (in Å)

Co	-0.585400000	-0.030298000	1.088724000
Co	-0.982975000	0.017556000	-1.346070000
Co	-3.152543000	-1.212000000	-1.898506000
S	-2.633061000	-0.569064000	0.127072000
Co	-2.506293000	-1.324616000	2.176513000
Co	-2.757001000	1.191469000	1.543507000
Co	-3.170842000	1.254754000	-1.084123000
Rh	3.824178000	2.031496000	-0.049814000
Rh	3.890331000	-1.945066000	-0.106753000
C	4.358260000	2.656924000	2.013881000
C	5.457614000	3.015518000	1.138653000
C	4.977339000	3.989736000	0.211398000
C	3.585735000	4.240176000	0.515608000
C	3.213518000	3.447928000	1.647491000
C	4.223747000	-2.688736000	1.964790000
C	5.471829000	-2.783097000	1.229657000
C	5.292014000	-3.732683000	0.174572000
C	3.933820000	-4.211554000	0.247501000
C	3.284857000	-3.596785000	1.369861000
C	0.364173000	-0.162550000	2.541163000
C	-1.438856000	2.013411000	2.374707000
C	-2.411427000	-0.484721000	3.750815000
C	-3.010658000	2.599492000	0.262660000
C	-4.201413000	1.614377000	2.474275000
C	-4.845655000	1.653523000	-1.497566000
C	-4.764405000	-1.919850000	-1.617340000
C	-2.211392000	2.140293000	-2.270165000
C	-3.425806000	-0.337604000	-3.430672000
C	-2.199595000	-2.653192000	-2.340454000
C	-0.395707000	-0.000799000	-2.990185000
C	-1.410239000	-2.719807000	2.295835000
C	-4.101213000	-2.108654000	2.314642000
B	4.592628000	-0.858252000	-1.919813000
H	5.490566000	-1.309191000	-2.597639000
B	4.770179000	0.063721000	-0.389916000
H	5.920564000	0.078263000	0.009380000
B	4.559030000	1.023891000	-1.892670000
H	5.441851000	1.525051000	-2.554946000
B	2.017414000	0.940247000	-0.687283000
B	3.103659000	0.023544000	0.425087000
H	2.714146000	0.007999000	1.569163000
B	2.045636000	-0.897099000	-0.714234000
O	-5.804185000	-2.408816000	-1.470627000
O	-5.929182000	1.925695000	-1.809973000
O	-3.644833000	0.092191000	-4.487062000
O	-1.753840000	2.874147000	-3.050088000
O	-3.067562000	3.779094000	0.307057000
O	0.060190000	-0.054253000	-4.057209000
O	-5.124400000	1.902170000	3.115417000
O	-5.118906000	-2.648585000	2.434279000

O	-0.724179000	-3.643792000	2.452944000
O	-2.362919000	-0.102097000	4.846987000
O	1.001516000	-0.288485000	3.506821000
O	-0.748133000	2.732130000	2.982699000
O	-1.640880000	-3.605709000	-2.693035000
S	2.845957000	1.776337000	-2.173339000
S	2.906053000	-1.660297000	-2.222271000
S	0.283460000	1.548709000	-0.265042000
S	0.323827000	-1.562838000	-0.332829000
H	6.472625000	2.601022000	1.177759000
H	4.406168000	1.943231000	2.846046000
H	2.224789000	3.417745000	2.122854000
H	2.924738000	4.926197000	-0.030451000
H	5.555434000	4.455966000	-0.596379000
H	6.392096000	-2.229175000	1.452920000
H	4.041460000	-2.060060000	2.845358000
H	6.043774000	-4.028357000	-0.567708000
H	3.471937000	-4.930997000	-0.441689000
H	2.250157000	-3.765813000	1.693870000

References

1. Led, J.J.; Gesmar, H. Application of the linear prediction method to NMR spectroscopy. *Chem. Rev.* **1991**, *91*, 1413−1426.
2. Yang, L.; Simionescu, R.; Lough, A.; Yan, H. Some observations relating to the stability of the BODIPY fluorophore under acidic and basic conditions. *Dyes Pigm.* **2011**, *91*, 264−267.
3. Weiss, R.; Grimes, R. N. Sources of Line Width in Boron-11 Nuclear Magnetic Resonance Spectra. Scalar Relaxation and Boron-Boron Coupling in B_4H_{10} and B_5H_9. *J. Am. Chem. Soc.* **1978**, *100*, 1401−1405.
